# Invariant solutions, lie symmetry analysis, bifurcations and nonlinear dynamics of the Kraenkel-Manna-Merle system with and without damping effect

**DOI:** 10.1038/s41598-024-77833-5

**Published:** 2024-11-01

**Authors:** Khaled Aldwoah, Shabir Ahmad, Faez Alqarni, Jihad Younis, Hussam E. Hashim, Manel Hleili

**Affiliations:** 1 Department of Mathematics, Faculty of Science, Islamic University of Madinah, Madinah 42351, Saudi Arabia; 2 Department of Mathematics and Physics, University of Campania “Luigi Vanvitelli”, 81100 Caserta, Italy; 3 Department of General Studies, University of Prince Mugrin (UPM), 42311 Madinah, Saudi Arabia; 4 Department of Mathematics, Aden University, Aden, Yemen; 5 Department of Mathematics, Turabah University College, Taif University, P.O. Box 11099, 21944 Taif, Saudi Arabia; 6 Department of Mathematics, Faculty of Science, University of Tabuk, P.O. Box 741, 71491 Tabuk, Saudi Arabia

**Keywords:** Nonlinear systems, Phase portraits, Ferromagnetic materials, Lie group transformation, Bifurcations, Chaos, Mathematics and computing, Applied mathematics

## Abstract

This work investigates the Kraenkel-Manna-Merle (KMM) system, which models the nonlinear propagation of short waves in saturated ferromagnetic materials subjected to an external magnetic field, despite the absence of electrical conductivity. The study aims to explore and derive new solitary wave solutions for this system using two distinct methodological approaches. In the first approach, the KMM system is transformed into a system of nonlinear ordinary differential equations (ODEs) via Lie group transformation. The resulting ODEs are then solved analytically using a similarity invariant approach, leading to the discovery of various types of solitary wave solutions, including bright, dark, and exponential solitons. The second approach involves applying wave and Galilean transformations to reduce the KMM system to a system of two ODEs, both with and without damping effects. This reduced system is further analyzed to investigate its bifurcation behavior, sensitivity to initial conditions, and chaotic dynamics. The analysis reveals the presence of strange multi-scroll chaotic dynamics in the presence of damping and off-boosting dynamics without damping. In addition to these approaches, the study also applies the planar dynamical theory to obtain further new soliton solutions of the KMM system. These solitons include bright, kink, dark, and periodic solutions, each of which has been visualized through 3D and 2D graphs. The results of this research provide new insights into the dynamics of the KMM system, with potential applications in magnetic data storage, magnonic devices, material science, and spintronics.

## Introduction

Nonlinear wave equations and complex physical phenomena inherently incorporate nonlinear and integrated dispersive effects. This nonlinearity arises from the connection between higher amplitude waves amplifying alongside lower amplitude waves. Due to nonlinearity and dispersion effect, the most stable wave is generated which is termed as soliton. The soliton theory has several applications in nonlinear sciences^1,2^. To find soliton solutions of nonlinear evolution equations, various methods have been introduced in the literature. For instance, F-expanison method^3,4^, Kudrayshov method^5^, Darboux transformation^6,7^, exp($-\Phi (\xi )$) method^8^, Hirota bilinear method^9,10^, generalized exponential rational function method^11^, logarithmic transformations^12^, complete discrimination system^13^, the Cole-Hopf transformation^14^, and many more^16–19^.

With the remarkable advancement in information technology aimed at fulfilling the demands for extensive data and high-density storage, there has been a surge of intriguing analysis on ferromagnetic materials in recent decades. Thanks to recent technological strides, it is now feasible to fabricate minuscule ferromagnetic particles. Understanding the characteristics of micro- and super micro- structures in nanoscale ferrous metals is imperative. In magnetization scenarios like these, tiny nano-particles can be regarded as homogeneous across the particles, represented by a magnetic moment. The dipolar movements of these magnetic moments enable communication among ferromagnetic particles, leading to the continuous generation of solitons through their interactions. Consequently, a broad spectrum of phenomena related to the dissemination of solitary waves has been investigated^20–22^. So, as interest grows in advancing large-scale information storage and transmission through electromagnetic materials, the study of solitons has become increasingly vital for microstructures and nanoscale phenomena in ferromagnetic media.

In the literature^23^, Kraenkel et al. examined the propagation of short waves in saturated ferromagnetic materials with zero conductivity under the influence of an external field. They formulated the subsequent nonlinear evolution system as:

1 $$\begin{aligned} {\left\{ \begin{array}{ll} \textsf{v}_{xt}-\textsf{z}_{x}\textsf{v}+k_1\textsf{v}_x=0, \\ \textsf{z}_{xt}-\textsf{v}\textsf{v}_{x}=0{,} \end{array}\right. } \end{aligned}$$

where $\textsf{v}=\textsf{v}(x,t)$ denotes the magnetization, $\textsf{z}=\textsf{z}(x,t)$ provides the external magnetic field and $k_1$ represents damping coefficient. The KMM system can explain the nonlinear propagation of short waves in saturated ferromagnetic materials with zero conductivity. The considered system (1) has a Lax pair and is integrable when the damping coefficient $k_1=0$. Several studies have been conducted to find various types of soliton solutions of KMM. For instance, generalized $\frac{G^{'}}{G}$-expansion method was utilized to analyze hump, loop, cusp, and kink solitons of KMM system^24^. In literature^25^, auxiliary equation method to KMM system to find loop-like soliton. Some other studies on KMM system includes^26–28^.

Lie symmetry analysis (LSA) is used to analyze nonlinear differential equations and is based on the concept of symmetries, which are the transformations that leave the equation invariant. The process of Lie symmetry analysis and finding invariant solutions is frequently utilized in various branches of physics and engineering, including fluid dynamics, solid mechanics, quantum mechanics, and nonlinear optics, among others^29–31^. It gives valuable insights into the dynamics of nonlinear systems and helps in understanding their underlying symmetries and structures. There are several nice works on LSA. For instance, Luo et al. analyzed conservation laws, LSA and some solitary waves of nonlinear symmetric regularized long-wave (SRLW) equation^32^. Guan et al. analysed LSA and travelling waves solutions of Cahn-Allen equation. Kumar et al. studied LSA and solitary waves for coupled breaking soliton model^33^. LSA and invaraint solutions of (2+ 1)-dimensional Bogoyavlensky-Konopelchenko equation with variable coefficients has been investigated by Kumar and his coauthors in the literature^34^. Invariant solutions, LSA and some soliton solutions of (2+ 1)-dimensional Riemann wave model and higher-dimensional modified dispersive water wave system have been reported in literature^35,36^. Some other studies can be found in^37–39^.

The theory of dynamical systems helps in understanding the behavior of these systems, including their long-term dynamics, stability properties, and the occurrence of bifurcations. Bifurcations provide the qualitative behavior of the dynamical systems such as stability, periodicity, or chaos. Phase portraits are useful for visualizing dynamics of two-dimensional system where the state of the system can be represented by a pair of variables. The study of dynamical systems, bifurcation, and phase portraits provides valuable insights into the evolution of complex systems^29^. Some works on the dynamical aspects and soliton solutions of nonlinear evolution equations are listed in^42–44^.

Since KMM system have several applications in ferromagnetic materials. Various results on KMM system have been published in the literature. However, invariant solution, LSA, dynamical properties and some more advanced soliton solutions via direct method have not previously reported. To fill this gap, we have used these technique to investigate KMM system. This article examines the LSA, which is a potent approach for resolving and interpreting differential equations. It is common practice to convert nonlinear partial differential equations (NLPDEs) into ODEs using the Lie symmetry approach. The ODE can then be solved analytically or numerically using similarity invariant. Furthermore, the KMM system for both cases i.e., $k_1\ne 0$ and $k_1=0$, the bifurcation, complex dynamics, and new soliton solutions in terms of Jacobi function are analyzed and depicted via 3D graphs.

## Lie symmetry analysis

**Fig. 1:**
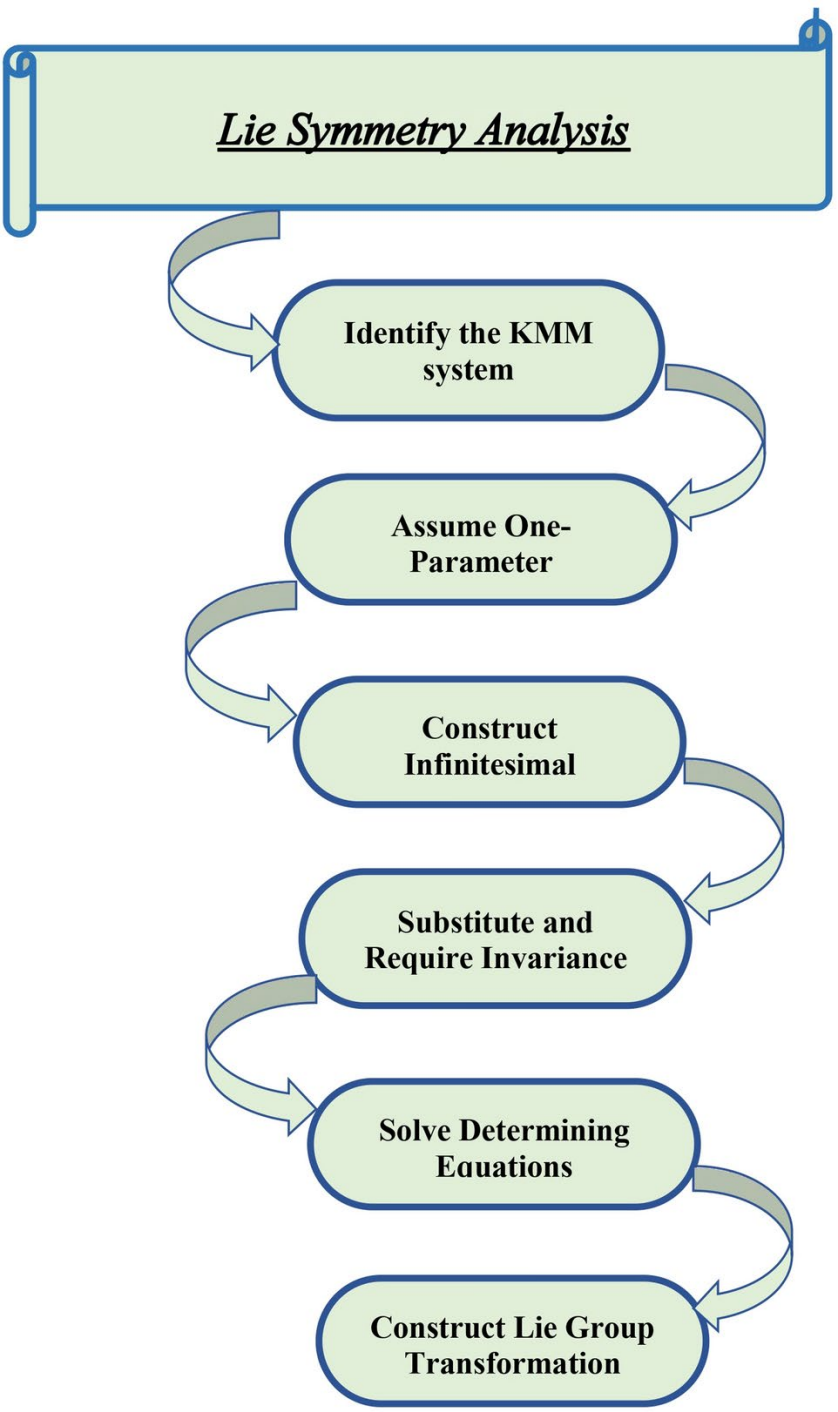
Steps of performing the classical Lie symmetry analysis with one-parameter transformation.

This section examines one-parameter Lie group transformation using the Lie symmetry analysis approach for Eq. (1). The Fig.1 illustrates the overview of extracting the results using classical Lie symmetry analysis. The following transformation is carried out using the infinitesimal transformation and the basic formula described below, which is used to acquire Eq. (2).

$$\begin{aligned} x^i\rightarrow x^i+\epsilon \xi ^i(x). \end{aligned}$$

The conventional Lie symmetry methodology, which uses an infinitesimal transformation to find differential equation invariants or conserved quantities, leads to this conclusion. The original system is reduced or invariant solutions are obtained using these changes.

2 $$\begin{aligned} {\left\{ \begin{array}{ll} \textsf{x} \rightarrow x +\epsilon ~ \varpi ^{1}(x,t,\textsf{v},\textsf{z})+O(\epsilon ^2),\\ \textsf{t} \rightarrow {t} +\epsilon ~ \varpi ^{2}(x,t,\textsf{v},\textsf{z})+O(\epsilon ^2),\\ \textsf{v} \rightarrow \textsf{v} +\epsilon ~ \zeta ^{1}(x,t,\textsf{v},\textsf{z})+O(\epsilon ^2),\\ \textsf{z} \rightarrow \textsf{z} +\epsilon ~ \zeta ^{2}(x,t,\textsf{v},\textsf{z})+O(\epsilon ^2), \end{array}\right. } \end{aligned}$$

with the tiny parameter $\epsilon<<~1$ , in the case of a one-parameter Lie group transformation, where the variables are $x,~t,~\textsf{v}~\text {and}~\textsf{z}$, Eq. (2) represents a generic form of classical Lie symmetry. The specific form of the infinitesimals is $\omega ^1,~\omega ^2,~\zeta ^1~\text {and}~\zeta ^2$, and the Lie symmetry analysis establishes it. Through this approach, the differential equation’s underlying symmetries become visible, which can be utilized to simplify the issue or provide analytical solutions. Its generator, a tiny form, is

3 $$\begin{aligned} \mathcal {G}= \varpi ^{1}(x,t,\textsf{v},\textsf{z})\frac{\partial }{\partial x}+\varpi ^{2}(x,t,\textsf{v},\textsf{z})\frac{\partial }{\partial t}+\zeta ^{1}(x,t,\textsf{v},\textsf{z})\frac{\partial }{\partial \textsf{v}}+\zeta ^{2}(x,t,\textsf{v},\textsf{z})\frac{\partial }{\partial \textsf{z}}. \end{aligned}$$

The symmetry generator is extended to the derivatives of $\textsf{v}$ and $\textsf{z}$ that are found in the nonlinear systems by the prolongation. The extended generator, for instance, acts on it and concerning the infinitesimal generator, its prolongation is

4 $$\begin{aligned} \text {pr}^{1} \mathcal {G}=\mathcal {G}+\zeta _x^{\textsf{v}}\frac{\partial }{\partial \textsf{v}_x}+\zeta _x^{\textsf{z}}\frac{\partial }{\partial \textsf{z}_x}+\zeta _{xt}^{\textsf{v}}\frac{\partial }{\partial \textsf{v}_{xt}}+\zeta _{xt}^{\textsf{z}}\frac{\partial }{\partial \textsf{z}_{xt}}. \end{aligned}$$

Where

5 $$\begin{aligned} {\left\{ \begin{array}{ll} \zeta _x^1= \textsf{D}_x(\zeta ^1)-\textsf{v}_x\textsf{D}_x(\varpi ^1)-\textsf{v}_t\textsf{D}_x(\varpi ^2),\\ \zeta _x^2= \textsf{D}_x(\zeta ^2)-\textsf{z}_x\textsf{D}_x(\varpi ^1)-\textsf{z}_t\textsf{D}_x(\varpi ^2),\\ \zeta _{xt}^1= \textsf{D}_x\textsf{D}_t(\zeta ^1-\varpi ^1\textsf{v}_x-\varpi ^2\textsf{v}_t)+\varpi ^1\textsf{v}_{xxt}+\varpi ^2\textsf{v}_{xtt},\\ \zeta _{xt}^2= \textsf{D}_x\textsf{D}_t(\zeta ^1-\varpi ^1\textsf{z}_x-\varpi ^2\textsf{z}_t)+\varpi ^1\textsf{z}_{xxt}+\varpi ^2\textsf{z}_{xtt}, \end{array}\right. } \end{aligned}$$

as $\textsf{D}_x,~\textsf{D}_t$ denotes a total derivatives with respect to *x* and *t*. Based on the requirements of invariance, the following formulas could be expressed as

6 $$\begin{aligned} {\left\{ \begin{array}{ll} \text {pr}^{1}\mathcal {G}(\Delta _1)|_{\Delta _1=0}=0,\\ \text {pr}^{1}\mathcal {G}(\Delta _2)|_{\Delta _2=0}=0, \end{array}\right. } \end{aligned}$$

where

7 $$\begin{aligned} \Delta _1=\textsf{v}_{xt}-\textsf{z}_{x}\textsf{v}-k_1\textsf{v}_x,\quad \quad \Delta _2=\textsf{z}_{xt}-\textsf{v}_{x}\textsf{z}. \end{aligned}$$

Next, the following is the system of over-determined equations:

8 $$\begin{aligned} {\left\{ \begin{array}{ll} \varpi _t^1=\varpi _{\textsf{v}}^1=\varpi _{\textsf{z}}^1=0,\\ \varpi _tt^2=\varpi _{\textsf{v}}^2=\varpi _{\textsf{z}}^2=0,\\ \zeta _{\textsf{v}}^1=-\varpi _{t}^2,\quad \zeta _{\textsf{z}}^1=\zeta _x^1=0,\\ \zeta _t^2=\zeta _{\textsf{v}}^2=\zeta _x^2=0,\\ \zeta _{\textsf{z}}^2=\frac{\zeta _2}{\textsf{z}}. \end{array}\right. } \end{aligned}$$

When this system is solved, we get

9 $$\begin{aligned} \varpi ^1=\mathcal {F}_1(x), \quad \varpi ^2=c_1t+c_2,\quad \zeta _1=-c_1\textsf{v}+\mathcal {F}_2(x),\quad \zeta ^2=c_3\textsf{z}, \end{aligned}$$

where $\mathcal {F}_1(x)$ and $\mathcal {F}_2(x)$ are arbitrary functions and $c_1$, $c_2$ and $c_3$ are arbitrary constants. Simply appropriate can be obtained by taking $\mathcal {F}_1(x)=c_4$ and $\mathcal {F}_2(x)=c_5$ and substituting them as shown above.

10 $$\begin{aligned} \varpi ^1=c_4, \quad \varpi ^2=c_1t+c_2,\quad \zeta _1=-c_1\textsf{v}+c_5,\quad \zeta ^2=c_3\textsf{z}, \end{aligned}$$

where the arbitrary constants are $c_1$, $c_2$, $c_3$, $c_4$ and $c_5$. Consequently, the generators listed below span Lie algebra $\mathcal {L}_4$ of infinitesimal symmetries.

11 $$\begin{aligned} {\left\{ \begin{array}{ll} \mathcal {G}_1=\frac{\partial }{\partial x},\\ \mathcal {G}_2=\frac{\partial }{\partial t}\\ \mathcal {G}_3=\textsf{z}\frac{\partial }{\partial \textsf{z}},\\ \mathcal {G}_4=\textsf{v}\frac{\partial }{\partial \textsf{v}}. \end{array}\right. } \end{aligned}$$

A classification tool that effectively arranges subalgebras is called an optimum system for a Lie algebra^40^. In particular, it offers a limited collection of subalgebras from which one subalgebra can be conjugated with another using the original algebra’s symmetries. The discussion of invariant solutions to control systems with symmetry reductions is discussed in subsection. Now, the commutator table $[\mathcal {G}_i,\mathcal {G}_j]$ and adjoint table ${\textit{Ad}(exp(\epsilon \mathcal {G}_i),\mathcal {G}_j)}$ for generators of symmetry as shown in Tables 1 and 1. The algebras structure is determined by the commutation relations among the generators:

$$\begin{aligned} {[}\mathcal {G}_i,\mathcal {G}_j]=\mathcal {G}_i\mathcal {G}_j-\mathcal {G}_j\mathcal {G}_i, \end{aligned}$$

where $\mathcal {G}_i$ and $\mathcal {G}_j$ are symmetry generators. The set of symmetry generators spans the Lie algebra. We will explicitly determine these relations to compute the commutator table. We calculate the activity of one generator on another to create the adjoint table.

$$\begin{aligned} Ad(exp(\epsilon \mathcal {G}_i))\mathcal {G}_j=\mathcal {G}_j+\epsilon [\mathcal {G}_i,\mathcal {G}_j]+\frac{\epsilon ^2}{2}[\mathcal {G}_i,[\mathcal {G}_i,\mathcal {G}_j]]+... \end{aligned}$$

**Table 1:** Symmetry generators for a table of commutators are.

[$\mathcal {G}_i,\mathcal {G}_j$]	$\mathcal {G}_1$	$\mathcal {G}_2$	$\mathcal {G}_3$	$\mathcal {G}_4$
$\mathcal {G}_1$	0	0	0	0
$\mathcal {G}_2$	0	0	0	0
$\mathcal {G}_3$	0	0	0	0
$\mathcal {G}_4$	0	0	0	0

**Table 2:** Symmetry generators for a table of adjoint are.

${\textit{Ad}(exp(\epsilon \mathcal {G}_i),\mathcal {G}_j)}$	$\mathcal {G}_1$	$\mathcal {G}_2$	$\mathcal {G}_3$	$\mathcal {G}_4$
$\mathcal {G}_1$	$\mathcal {G}_1$	$\mathcal {G}_2$	$\mathcal {G}_3$	$\mathcal {G}_4$
$\mathcal {G}_2$	0	$\mathcal {G}_2$	$\mathcal {G}_3$	$\mathcal {G}_4$
$\mathcal {G}_3$	$\mathcal {G}_1$	$\mathcal {G}_2$	$\mathcal {G}_3$	0
$\mathcal {G}_4$	$\mathcal {G}_1$	$\mathcal {G}_2$	0	$\mathcal {G}_4$

### Invariant solutions and Symmetry reductions

Extract invariant solutions for all clusters using symmetry generators discussed in Eq. (11). For this, let $\Gamma _1=\mathcal {G}_1.$

For cluster 1, the characteristic equation is in the following form

12 $$\begin{aligned} \frac{dx}{1}=\frac{dt}{0}=\frac{d\textsf{v}}{0}=\frac{d\textsf{z}}{0}. \end{aligned}$$

The solution of the above characteristic equation for a defined variable is

13 $$\begin{aligned} \textsf{v}=\mathcal {R}_1(\zeta ^1), \quad \quad \textsf{z}=\mathcal {R}_2(\zeta ^2), \end{aligned}$$

with $\zeta =(x+t)=\zeta ^1=\zeta ^2$. Convert Eq. (1) into a nonlinear ODEs by using Eq. (13).

14 $$\begin{aligned} {\left\{ \begin{array}{ll} \mathcal {R}_1^{''}-\mathcal {R}_2^{'}\mathcal {R}_1+K1\mathcal {R}_1^{'}=0,\\ \mathcal {R}_2^{''}-\mathcal {R}_1^{'}\mathcal {R}_1=0{.} \end{array}\right. } \end{aligned}$$

Solving the system of NLODEs with the help of NDsolve command of Mathematica yields the following set of solutions.

15 $$\begin{aligned}&\mathcal {R}_1(\zeta )=j_1 exp[\text {tanh}(\zeta )+j_2], \end{aligned}$$

16 $$\begin{aligned}&\mathcal {R}_2(\zeta )=\frac{\sqrt{1-exp[\text {sin}(\zeta )]}}{j_1}(j_1 \text {sech}(\zeta )+j_2)^2{.} \end{aligned}$$

Inserting these solutions into Eq. (1), to get required soliton solutions.

17 $$\begin{aligned} & \textsf{v}_{1,1}=\frac{j_1 exp[\text {tanh}(\zeta )+j_2[-\sqrt{1-exp[sin(\zeta )]}(j_1 \text {sech}(\zeta )+j_2]]}{j_1}, \end{aligned}$$

18 $$\begin{aligned} & \textsf{z}_{1,1}=e^{(j_1(\zeta ))}\times \frac{j_1exp[\tanh (\zeta )+j_2[-\sqrt{1-exp[sin(\zeta )]}(j_1 \text {sech}(\zeta )+j_2]]}{j_1}. \end{aligned}$$

**Fig. 2:**
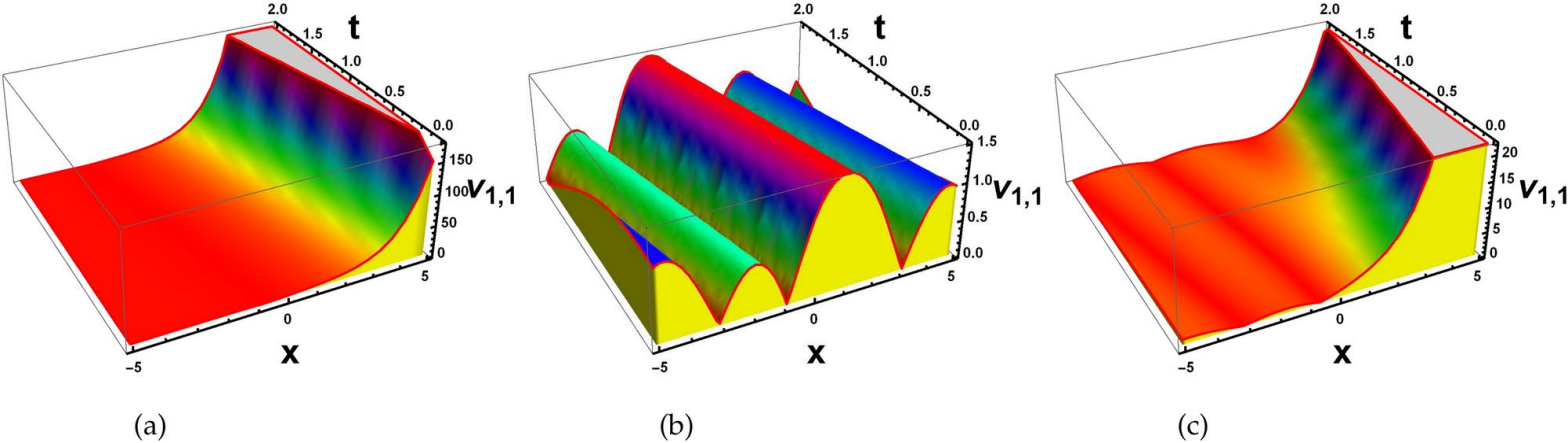
The depiction of 3D diagrams of $\textsf{v}_{1,1}$ illustrating the combo dark, bright solitons, periodic and exponential solutions under the different parametric values.

**Fig. 3:**
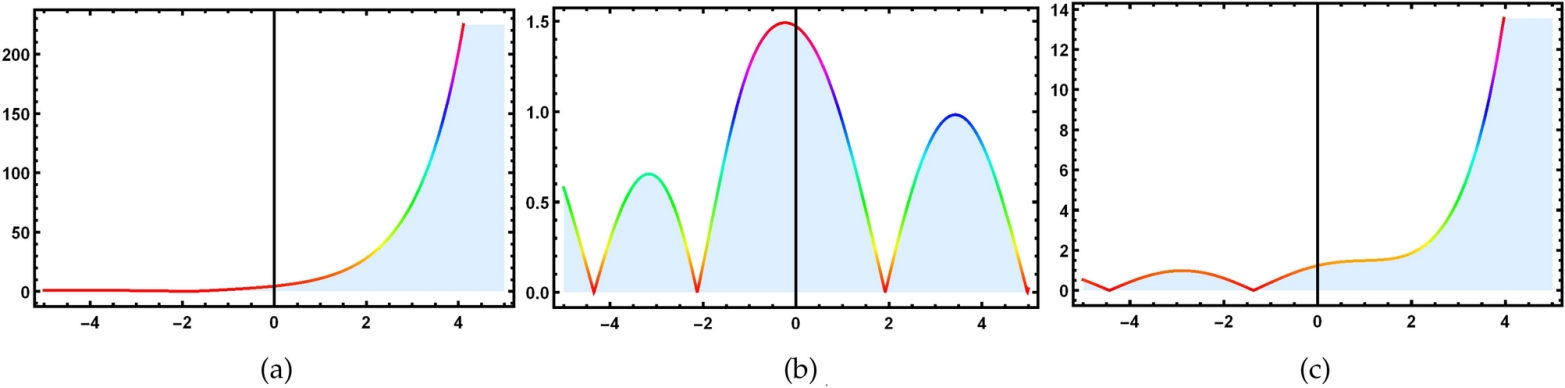
The depiction of 2D diagrams of $\textsf{v}_{1,1}$ illustrating the combo dark, bright solitons, periodic and exponential solutions under the different parametric values at $t=1.3.$.

**Fig. 4:**
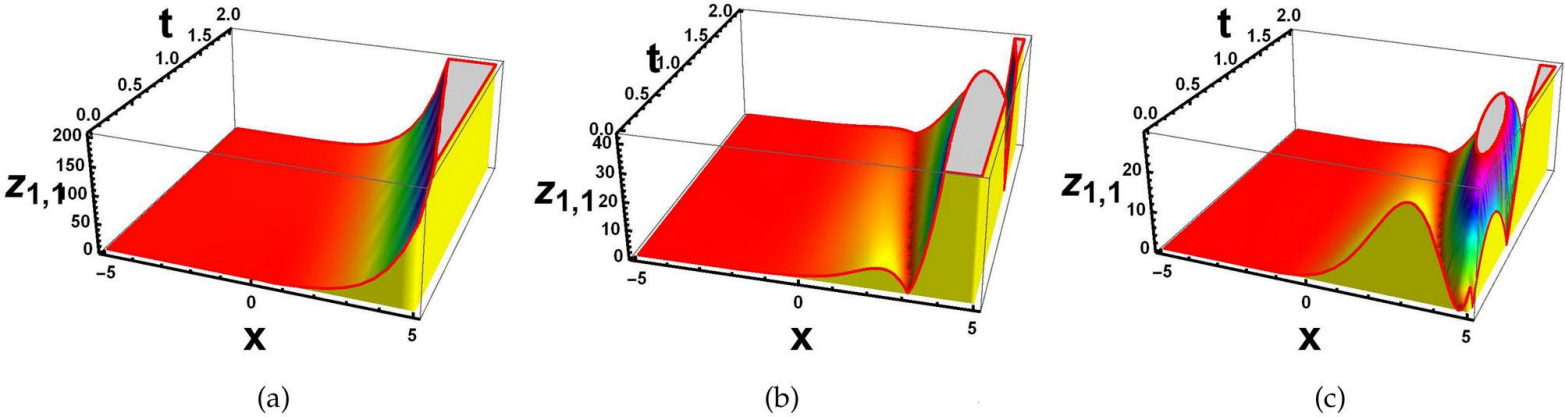
The depiction of 3D diagrams of $\textsf{z}_{1,1}$ illustrating the combo of dark, bright solitons, periodic and exponential solutions under the different parametric values.

**Fig. 5:**
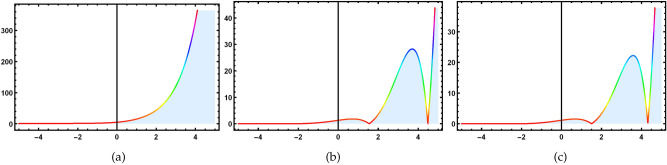
The depiction of 2D diagrams of $\textsf{z}_{1,1}$ illustrating the combo of dark, bright solitons, periodic and exponential solutions under the different parametric values at $t=1.5.$.

Since the $j_k$ where $k=1,2,..$ are all constants and $\zeta$ is function of *x* and *t*. Figs. (2, 3, 4 and 5), represent the combo of soliton solutions of dark, bright, periodic, and exponential solutions of equations 17 and 18.

## Dynamical behaviors of the KMMS with damping effect

To get the wave equation of KMMS (1), we utilize the wave transformation as:

19 $$\begin{aligned} {\left\{ \begin{array}{ll} \textsf{v}\left( x,t\right) =\mathcal {V}\left( \delta \right) ,\\ \textsf{z}\left( x,t\right) =\mathcal {Z}\left( \delta \right) , \end{array}\right. } \end{aligned}$$

where $\delta =\Lambda x+\sigma t$. Inserting Eq.(19) into Eq.(1), we derived the following system of nonlinear ODEs.

20 $$\begin{aligned} {\left\{ \begin{array}{ll} \sigma \mathcal {V}^{\prime \prime }- \mathcal {V}\mathcal {Z}^{\prime }+k1\mathcal {V}^{\prime }=0,\\ \sigma \mathcal {Z}^{\prime \prime }- \mathcal {V}\mathcal {V}^{\prime }=0. \end{array}\right. } \end{aligned}$$

After integrating the second equation in (20) once, we obtain the following result.

21 $$\begin{aligned} \mathcal {Z}^{\prime }=\frac{\mathcal {V}^{2}}{2\sigma }+\frac{\mathcal {C}}{\sigma }, \end{aligned}$$

where $\mathcal {C}$ portrays an integration constant. Via inserting Eq. (21) into the first equation in (20), one reach:

22 $$\begin{aligned} \mathcal {V}^{\prime \prime }-\frac{1}{2\sigma ^{2}} \mathcal {V}^{3}-\frac{\mathcal {C}}{\sigma ^{2}} \mathcal {V}+\frac{k1}{\sigma } \mathcal {V}^{\prime }=0. \end{aligned}$$

After the implementation of the Galilean transform, the Eq. (22) converted to a system of two ODEs, which are presented below:

23 $$\begin{aligned} {\left\{ \begin{array}{ll} \frac{d\mathcal {V}\left( \delta \right) }{d\delta }=\mathcal {W}(\delta ), \\ \frac{d\mathcal {W}\left( \delta \right) }{d\delta }=\mathcal {F}_1\mathcal {V}^{3}\left( \delta \right) +\mathcal {F}_2\mathcal {V}(\delta )-\mathcal {F}_3\mathcal {W}(\delta ), \end{array}\right. } \end{aligned}$$

where $$\mathcal {F}_1=\frac{1}{2\sigma ^{2}}, \mathcal {F}_2=\frac{\mathcal {C}}{\sigma ^{2}}$$ and $$\mathcal {F}_3=\frac{k1}{\sigma }$$.

### Stationary points and bifurcations

For the stationary points and bifurcations of the system (23), we refer the reader to^45^.

### Chaos in the governing model

In this part, we study the possibility of chaos in the system (23) by adding a perturbation. After adding perturbation, the following system is acquired:

24 $$\begin{aligned} {\left\{ \begin{array}{ll} \frac{d\mathcal {V}(t)}{dt}=\mathcal {W}(t), \\ \frac{d\mathcal {W}(t)}{dt}=\mathcal {F}_1\mathcal {V}^{3}(t)+\mathcal {F}_2\mathcal {V}(t)-\mathcal {F}_{3}\mathcal {W}+\omega \sin \left( \mu \textsf{t}\right) . \end{array}\right. } \end{aligned}$$

**Fig. 6:**
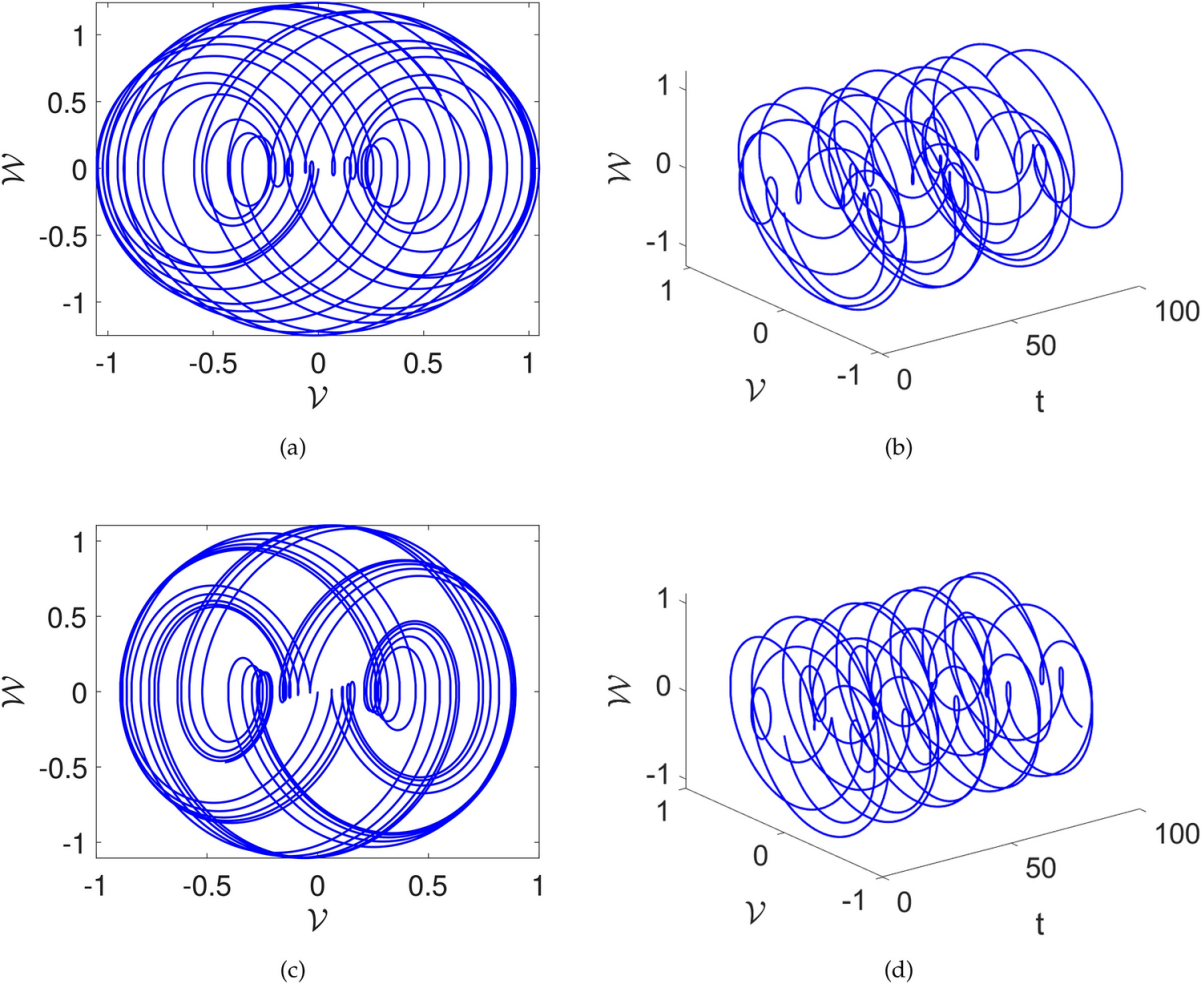
Chaotic visual depictions of a proposed system (23), while factoring in parameters as $\sigma =1,~C=-1,~k1=0.001$ and a,b)$\omega =1,~\mu =1$, c,d) $\omega =1,~\mu =2.1$ .

**Fig. 7:**
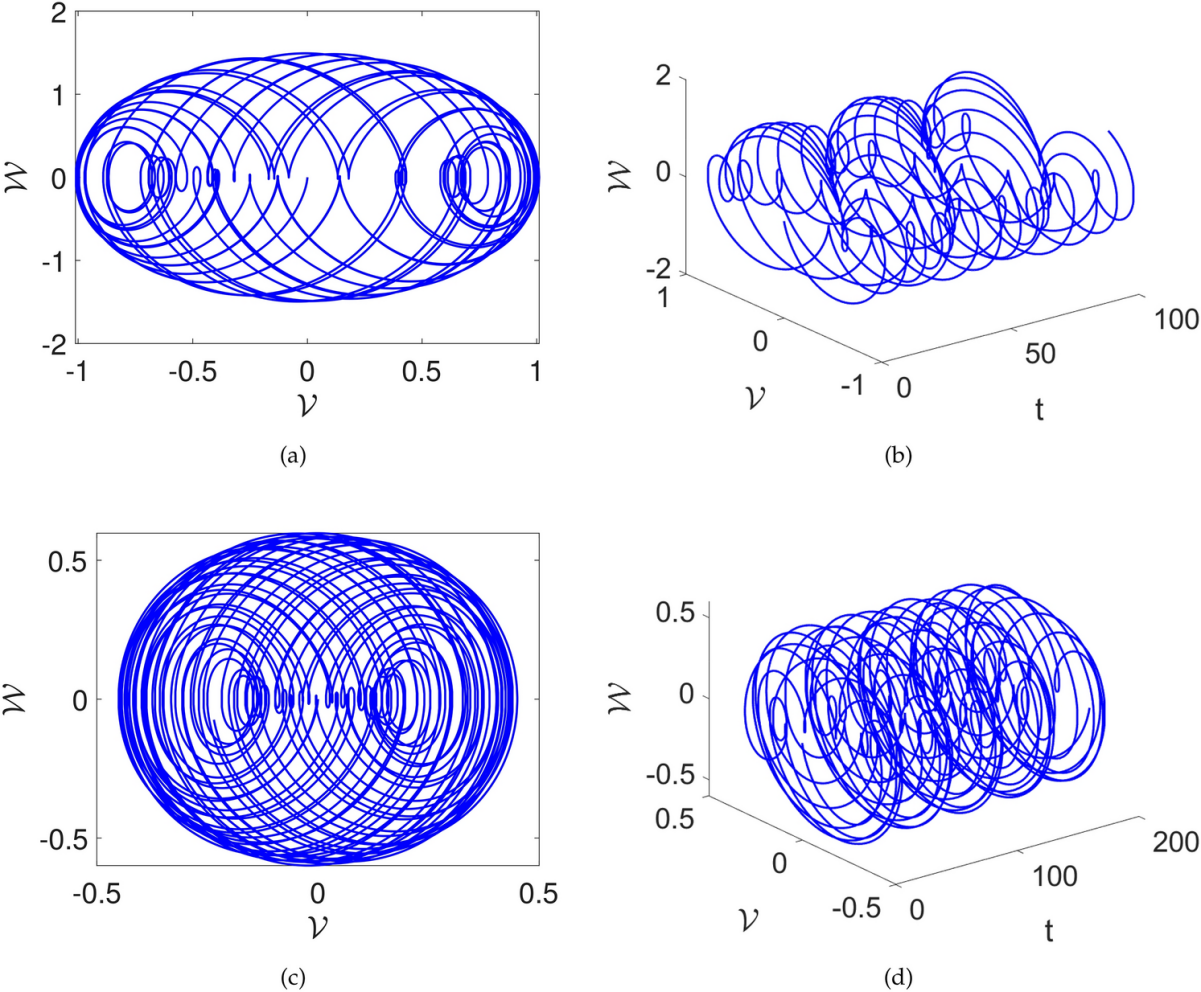
Chaotic visual depictions of a proposed system (23), while factoring in parameters as $\sigma =1,~C=-1,~k1=0.001$, and a,b)$\omega =2.1,~\mu =3.2$ and c,d) $\omega =0.5,~\mu =2.1$.

In Figs. 6 and 7, we explore the impact of the perturbed term $\omega \sin (\mu t)$ on the system (24). It’s important to note that $\omega$ gives amplitude and $\mu$ describes the frequency of the system.

The three-dimensional and two-dimensional vs time phase diagrams are presented. Upon scrutinizing the phase diagrams, one can see complex behavior. The $\omega$ and $\mu$ are varied and their influence can be seen in the Figs. 6 and 7. In Fig.6 the parameters are considered as $\sigma =1,~C=-1,~k1=0.001$ and the $\omega , \mu$ are considered as [(a),(b)] $\omega =1,~\mu =1$, [(c),(d)] $\omega =1,~\mu =2.1$. We see that there is multi-scroll dynamics with several oscillations with the proposed value. Furthermore, in Fig.7 the parameters are considered as $\sigma =1,~C=-1,~k1=0.001$ and the $\omega , \mu$ are considered as [(a),(b)] $\omega =2.1,~\mu =3.2$, [(c),(d)] $\omega =0.5,~\mu =2.1$. Here, also multi-scroll dynamics are observed. These outcomes highlight the sensitivity of the system’s dynamics to variations in the parameter $\mu$ and provide valuable insights into the effects of $\omega \sin (\mu t)$ on the dynamics of the considered system.

### Sensitivity analysis

Here, we study the influence of varying initial conditions on the dynamical evolution of the suggested model considered as:

25 $$\begin{aligned} {\left\{ \begin{array}{ll} \frac{d\mathcal {V}}{dt}=\mathcal {W}(t),\\ \frac{d\mathcal {W}}{dt}=\mathcal {F}_{1}\mathcal {V}^3(t)+\mathcal {F}_{2}\mathcal {V}(t)-\mathcal {F}_{3}\mathcal {W}(t). \end{array}\right. } \end{aligned}$$

**Fig. 8:**
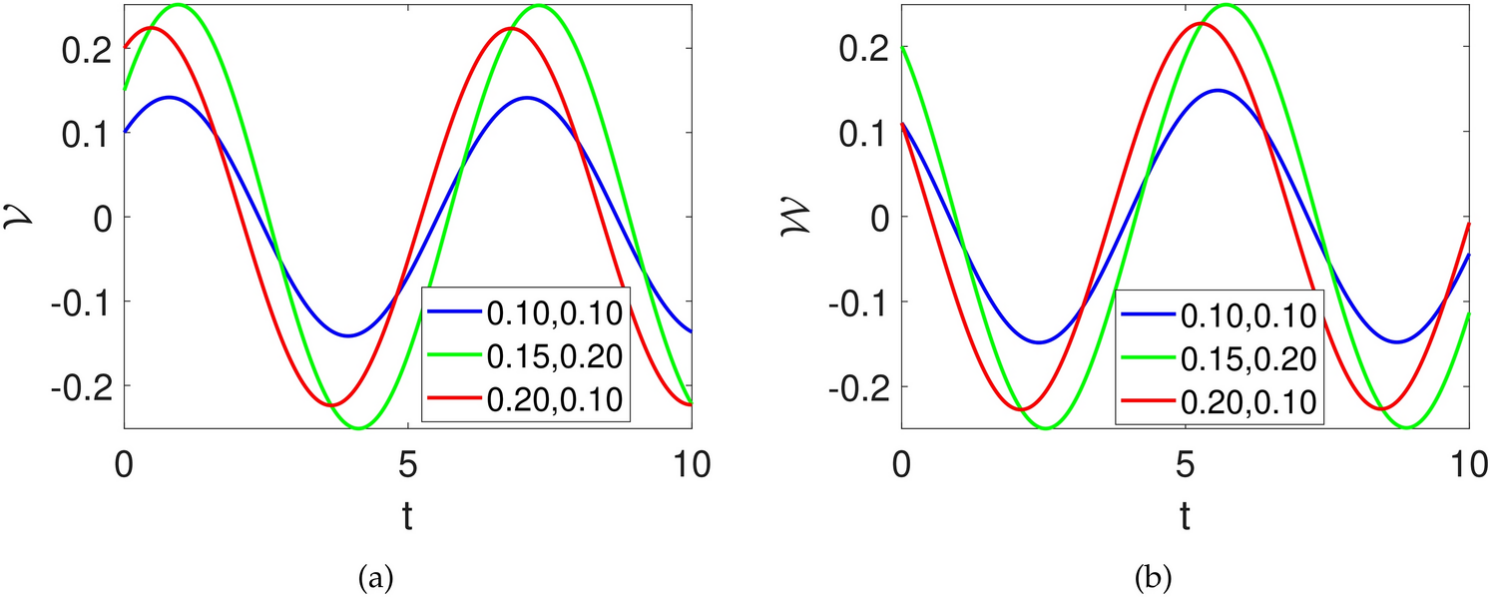
Numerical depiction of state variables of system (23) against time *t* with parameters utilized as $\sigma =1,~C=-1,~k1=0.001$ and initial values supposed as $[blue, (\mathcal {V}(0),\mathcal {W}(0))=(0.1,0)], [green, (\mathcal {V}(0),\mathcal {W}(0))=(0.3,0)],[red, (\mathcal {V}(0),\mathcal {W}(0))=(0.6,0)]$.

We consider several initial values to observe how much the system show changes in its dynamics. The initial conditions are supposed to be $[blue, (\mathcal {V}(0),\mathcal {W}(0))=(0.1,0)], [green, (\mathcal {V}(0),\mathcal {W}(0))=(0.3,0)],[red, (\mathcal {V}(0),\mathcal {W}(0))=(0.6,0)]$ while the parameters are considered as $\sigma =1,~C=-2$. The influence of varying initial values on the dynamics of system (25) is demonstrated in Fig.8.

## Dynamical behaviors of the KMMS without damping

Here, we consider the damping effect $k_1 = 0$ then from (22) we get:

26 $$\begin{aligned} \mathcal {V}^{\prime \prime }-\frac{1}{2\sigma ^{2}} \mathcal {V}^{3}-\frac{\mathcal {C}}{\sigma ^{2}} \mathcal {V}{.} \end{aligned}$$

After the implementation of the Galilean transform to Eq. (26), we get the following system:

27 $$\begin{aligned} {\left\{ \begin{array}{ll} \frac{d\mathcal {V}\left( \delta \right) }{d\delta }=\mathcal {W}(\delta ), \\ \frac{d\mathcal {W}\left( \delta \right) }{d\delta }=\mathcal {F}_1\mathcal {V}^{3}\left( \delta \right) +\mathcal {F}_2\mathcal {V}(\delta ), \end{array}\right. } \end{aligned}$$

where

$$\begin{aligned} \mathcal {F}_1=\frac{1}{2\sigma ^{2}}, \quad \quad \mathcal {F}_2=\frac{\mathcal {C}}{\sigma ^{2}}{.} \end{aligned}$$

### Stationary points and bifurcations

**Fig. 9:**
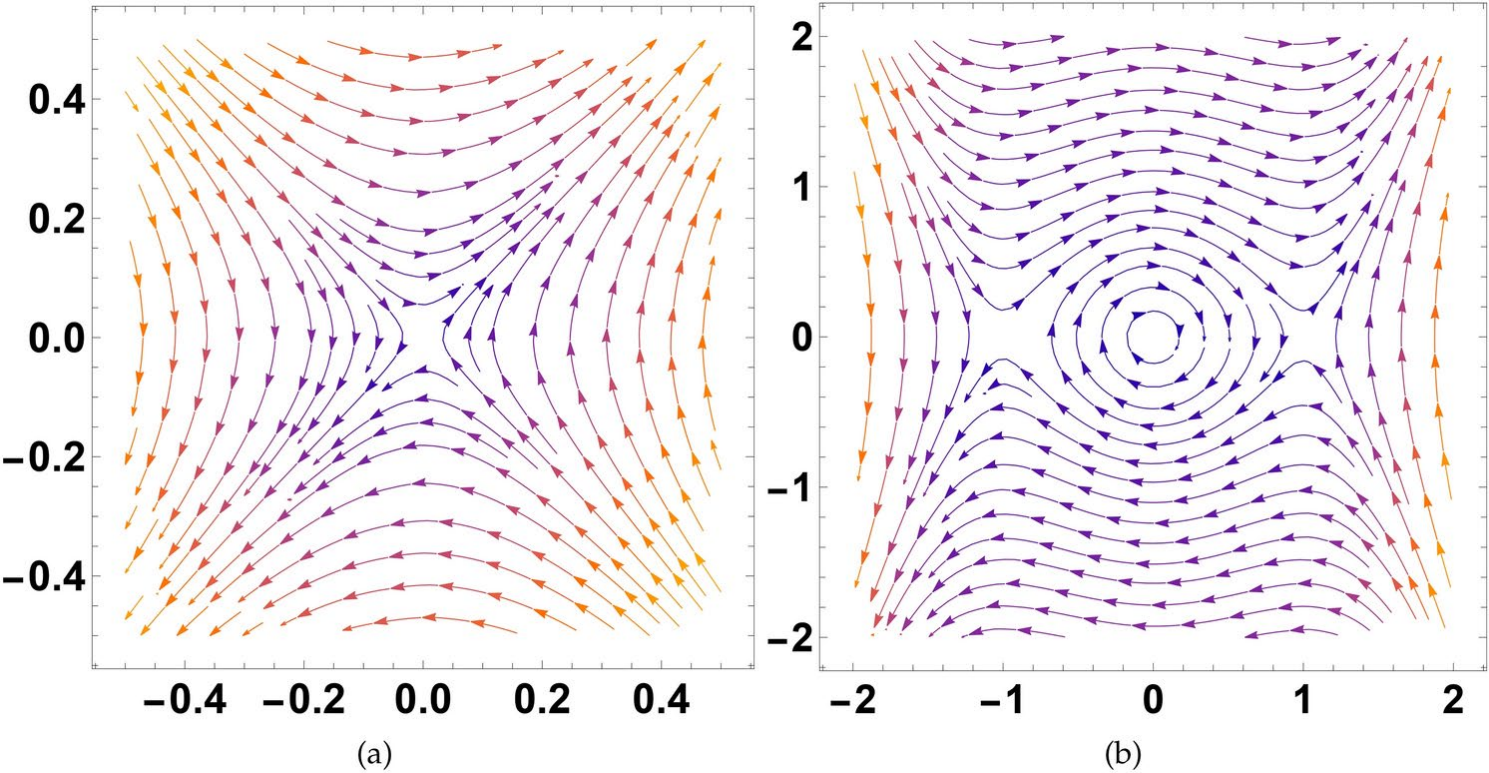
The depiction of phase diagrams illustrating the bifurcations of the introduced system (27) under varying conditions of $\mathcal {F}_{1}$, and $\mathcal {F}_{2}$ by using different parameters’ values.

In this section, we offer a detailed examination of the system outlined in Eq.(27). First, we have to compute equilibrium points and the bifurcation diagrams are presented in Fig.9. For this, we need to solve the system presented below:

$$\begin{aligned} {\left\{ \begin{array}{ll} \mathcal {W}=0{,} \\ \mathcal {F}_1\mathcal {V}^3+\mathcal {F}_2\mathcal {V}=0, \end{array}\right. } \end{aligned}$$

the calculated stationary points of system (27) are:

$$\begin{aligned} \mathcal {E}_1=\left( 0,0\right) ,\mathcal {E}_2=\left( -\iota \sqrt{\frac{\mathcal {F}_2}{\mathcal {F}_1}},0\right) ,\mathcal {E}_3=\left( \iota \sqrt{\frac{\mathcal {F}_2}{\mathcal {F}_1}},0\right) . \end{aligned}$$

The following is the determinant of the Jacobian matrix of the system (27).

$$\begin{aligned} \mathcal {D}\left( \mathcal {V},\mathcal {W}\right)= & {\begin{vmatrix}0&1\\3\mathcal {F}_1\mathcal {V}^2+\mathcal {F}_2&0\end{vmatrix}} =-3\mathcal {F}_1\mathcal {V}^2-\mathcal {F}_2. \end{aligned}$$

Case I: By considering the parameters in such a way that $\mathcal {F}_{1}>0, \mathcal {F}_{2}>0.$ This can be achieved by specifying the parameters as $\sigma =1,$ and $C=1$. Only one real SPs is observed which is (0, 0). From the figure, it can be seen that the point is a saddle, which can be observed from Fig.9a.

Case II: By choosing the parameters in such a way that $\mathcal {F}_{1}>0, \mathcal {F}_{2}<0.$ This can be obtained by selecting the parameters in the form $\sigma =1$ and $C=-1$. Three SPs are observed which are $(-1,0),(0,0),(1,0)$. The points $(-1,0)$ and (1, 0) are saddle points, while the point (0, 0) is center, which can be observed in Fig.9b.

### Chaos in the governing model (27)

In this part, we search for the possibility of chaos in the system (28) by adding a perturbed term. After the addition of perturbation, the following system is acquired:

28 $$\begin{aligned} {\left\{ \begin{array}{ll} \frac{d\mathcal {V}(t)}{dt}=\mathcal {W}(t), \\ \frac{d\mathcal {W}(t)}{dt}=\mathcal {F}_1\mathcal {V}^{3}(t)+\mathcal {F}_2\mathcal {V}(t)-\omega \sin \left( \mu \textsf{t}\right) . \end{array}\right. } \end{aligned}$$

**Fig. 10:**
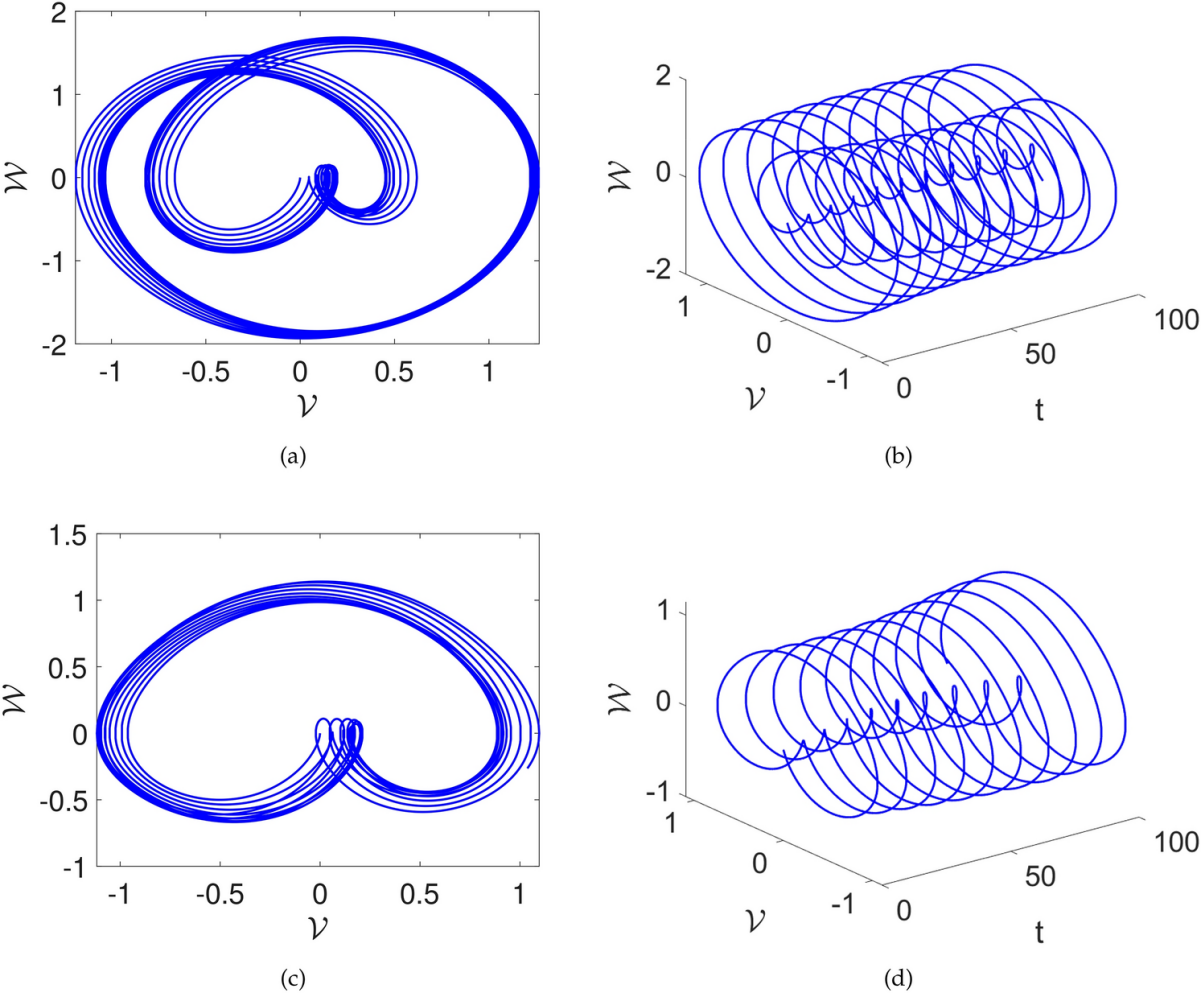
Chaotic visual depictions of a proposed system (28), while factoring in parameters as $\sigma =1,~C=-2$ and a,b)$\omega =1,~\mu =1$, c,d) $\omega =1,~\mu =2.1$ .

**Fig. 11:**
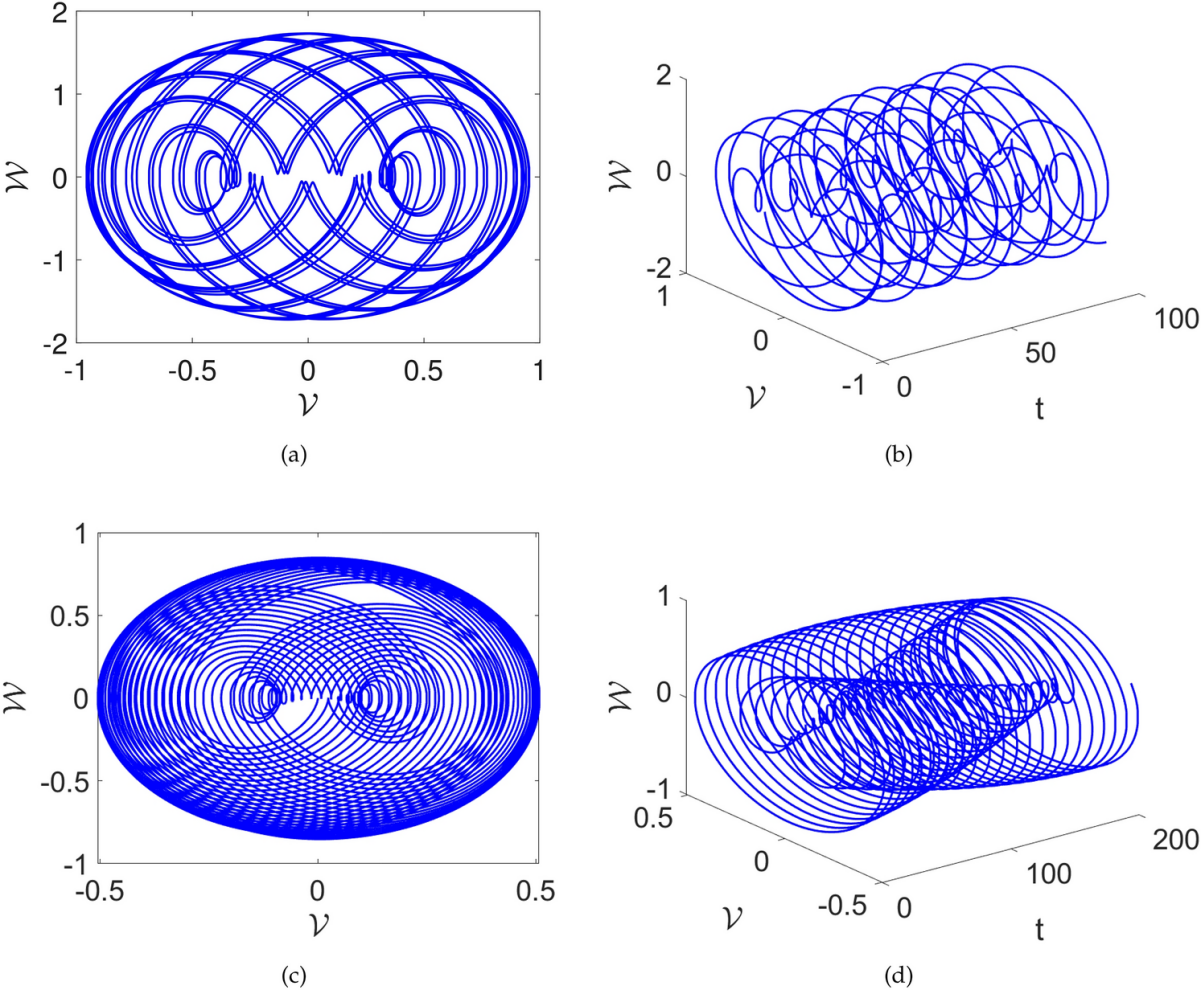
Chaotic visual depictions of a proposed system (28), while factoring in parameters as $\sigma =1,~C=-2$, and a,b)$\omega =2.1,~\mu =3.2$ and c,d) $\omega =0.5,~\mu =2.1$.

In Figs. 10 and 11, we investigate the impact of the perturbed term $\omega \sin (\mu t)$ on the system (28). Here also $\omega$ denotes amplitude and $\mu$ displays frequency of the system.

The two dimensional and two dimensional vs time phase diagrams are presented. Upon scrutinizing the phase diagrams, the complex dynamics are observed. The $\omega$ and $\mu$ are varied and their influence can be seen in the Fig. 10 and 11. In Fig.10 the parameters are considered as $\sigma =1,~C=-2$ and the $\omega , \mu$ are considered as [(a) and (b)] $\mu =1,~\omega =1$, [(c) and (d)] $\omega =1,~\mu =2.1$. We see that there are boosting-off and boosting dynamics of the proposed value. Furthermore, in Fig.11 the parameters are considered as $\sigma =1,~C=-2$ and the $\omega , \mu$ are considered as [(a) and (b)] $\omega =2.1,~\mu =3.2$, [(c) and (d)] $\omega =0.5,~\mu =2.1$. Here, also complex multi-scroll dynamics are observed. These findings visualize the effects and importance of the perturbed term $\omega \sin (\mu t)$ on the system (28).

### Sensitivity

Here, we analyze the influence of varying the initial conditions on the dynamical evolution of the suggested system considered as:

29 $$\begin{aligned} {\left\{ \begin{array}{ll} \frac{d\mathcal {V}}{dt}=\mathcal {W}(t),\\ \frac{d\mathcal {W}}{dt}=\mathcal {F}_{1}\mathcal {V}^3(t)+\mathcal {F}_{2}\mathcal {V}(t). \end{array}\right. } \end{aligned}$$

**Fig. 12:**
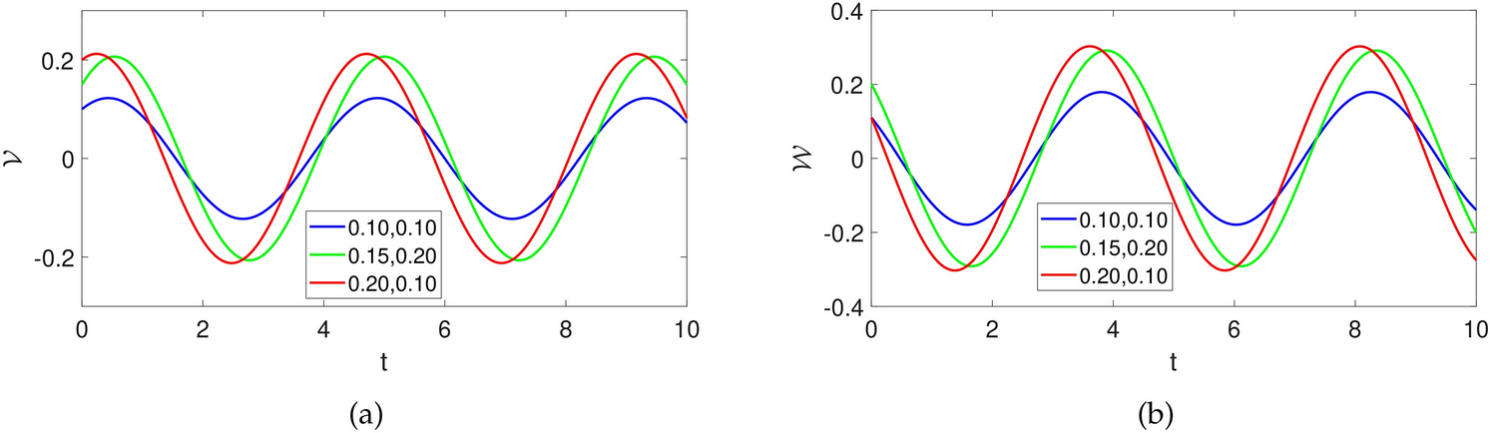
Numerical depiction of state variables of system (27) against time *t* with parameters utilized as $\sigma =1,~C=-2$ and initial values supposed as $[blue, (\mathcal {V}(0),\mathcal {W}(0))=(0.1,0)], [green, (\mathcal {V}(0),\mathcal {W}(0))=(0.3,0)],[red, (\mathcal {V}(0),\mathcal {W}(0))=(0.6,0)]$.

We consider several initial values to observe how much the system show changes in its dynamics. The initial conditions are supposed to be $[blue, (\mathcal {V}(0),\mathcal {W}(0))=(0.1,0)], [green, (\mathcal {V}(0),\mathcal {W}(0))=(0.3,0)],[red, (\mathcal {V}(0),\mathcal {W}(0))=(0.6,0)]$ while the parameters are considered as $\sigma =1,~C=-2$. The influence of varying initial values on the dynamics of system (27) is demonstrated in Fig.12.

## Kinks, bright and bright solitary waves of the KMMS with damping

Within this portion, we utilize the notions of planar dynamical system method to unveil different types of kinks, bright solitary waves, and dark waves originating from the KMMS Eq.(1) incorporating damping effects.

First, we have to construct the Hamiltonian function for Eq.(23) as follows:

$$\begin{aligned} \mathcal {H}\left( \mathcal {V},\mathcal {W}\right) = \frac{1+\mathcal {F}_3}{2}\mathcal {W}^2-\frac{\mathcal {F}_1\mathcal {V}^4}{4}-\frac{\mathcal {F}_2\mathcal {V}^2}{2}=\textsf{h}, \end{aligned}$$

here $\textsf{h}$ denotes Hamiltonian constant. Since $\mathcal {F}_1>0$ and $\mathcal {F}_2>0$

When $\textsf{h}$ $\in$ $\left( \frac{\mathcal {F}_{2}^{2}}{4\mathcal {F}_1},0\right)$, as a result, two periodic orbits emerge. Consequently, One can express the Hamiltonian function as follows:

30 $$\begin{aligned} \mathcal {W}^2= & \frac{\mathcal {F}_1}{2(1+\mathcal {F}_3)}\left( \mathcal {V}^4+\frac{2\mathcal {F}_2}{\mathcal {F}_1}\mathcal {V}^2+\frac{4\textsf{h}}{\mathcal {F}_1}\right) {,} \nonumber \\= & \frac{\mathcal {F}_1}{2(1+\mathcal {F}_3)}\left( \mathcal {V}^2-\upsilon _{1\textsf{h}^{2}}\right) \left( \upsilon _{2\textsf{h}^{2}}-\mathcal {V}^2\right) , \end{aligned}$$

where

$$\begin{aligned} \upsilon _{1\textsf{h}}=-\frac{\mathcal {F}_2}{\mathcal {F}_1}-\frac{\sqrt{\mathcal {F}^2_{2}-4\textsf{h}\mathcal {F}_1}}{\mathcal {F}_1},\quad \upsilon _{2\textsf{h}}=-\frac{\mathcal {F}_2}{\mathcal {F}_1}+\frac{\sqrt{\mathcal {F}^2_{2}-4\textsf{h}\mathcal {F}_1}}{\mathcal {F}_1}. \end{aligned}$$

The substitution of Eq. (30) into first part of system (23) and then integrating periodic orbits, we get

$$\begin{aligned} \int _{-\upsilon _{2\textsf{h}}}^{\mathcal {V}}\frac{d\eta }{\sqrt{\left( \mathcal {\eta }^2-\upsilon _{1\textsf{h}^{2}}\right) \left( \upsilon _{2\textsf{h}^{2}}-\mathcal {\eta }^2\right) }}= & \pm \sqrt{\frac{\mathcal {F}_1}{2(1+\mathcal {F}_3)}}\left( \delta -\delta _0\right) , \\ \int _{\mathcal {V}}^{\upsilon _{2\textsf{h}}}\frac{d\eta }{\sqrt{\left( \mathcal {\eta }^2-\upsilon _{1\textsf{h}^{2}}\right) \left( \upsilon _{2\textsf{h}^{2}}-\mathcal {\eta }^2\right) }}= & \mp \sqrt{\frac{\mathcal {F}_1}{2(1+\mathcal {F}_3)}}\left( \delta -\delta _0\right) . \end{aligned}$$

Because of the preceding equations, the solutions presented below can be acquired for the Jacobi function:

31 $$\begin{aligned} \textsf{v}_{1,2}= & \pm \upsilon _{2\textsf{h}}dn\left( \upsilon _{2\textsf{h}}\sqrt{\frac{\mathcal {F}_1}{2(1+\mathcal {F}_3)}}\left( \Lambda x+\sigma t-\delta _0\right) , \frac{\sqrt{\upsilon _{2\textsf{h}}^2-\upsilon _{1\textsf{h}}^2}}{\upsilon _{2\textsf{h}}}\right) , \end{aligned}$$

32 $$\begin{aligned} \textsf{z}_{1,2}= & \frac{1}{6\sigma }\left( \pm \upsilon _{2\textsf{h}}dn\left( \upsilon _{2\textsf{h}}\sqrt{\frac{\mathcal {F}_1}{2(1+\mathcal {F}_3)}}\left( \Lambda x+\sigma t-\delta _0\right) , \frac{\sqrt{\upsilon _{2\textsf{h}}^2-\upsilon _{1\textsf{h}}^2}}{\upsilon _{2\textsf{h}}}\right) \right) ^{3}\nonumber \\ & +\frac{\mathcal {C}}{\sigma }\left( \pm \upsilon _{2\textsf{h}}dn\left( \upsilon _{2\textsf{h}}\sqrt{\frac{\mathcal {F}_1}{2(1+\mathcal {F}_3)}}\left( \Lambda x+\sigma t-\delta _0\right) , \frac{\sqrt{\upsilon _{2\textsf{h}}^2-\upsilon _{1\textsf{h}}^2}}{\upsilon _{2\textsf{h}}}\right) \right) , \end{aligned}$$

**Fig. 13:**
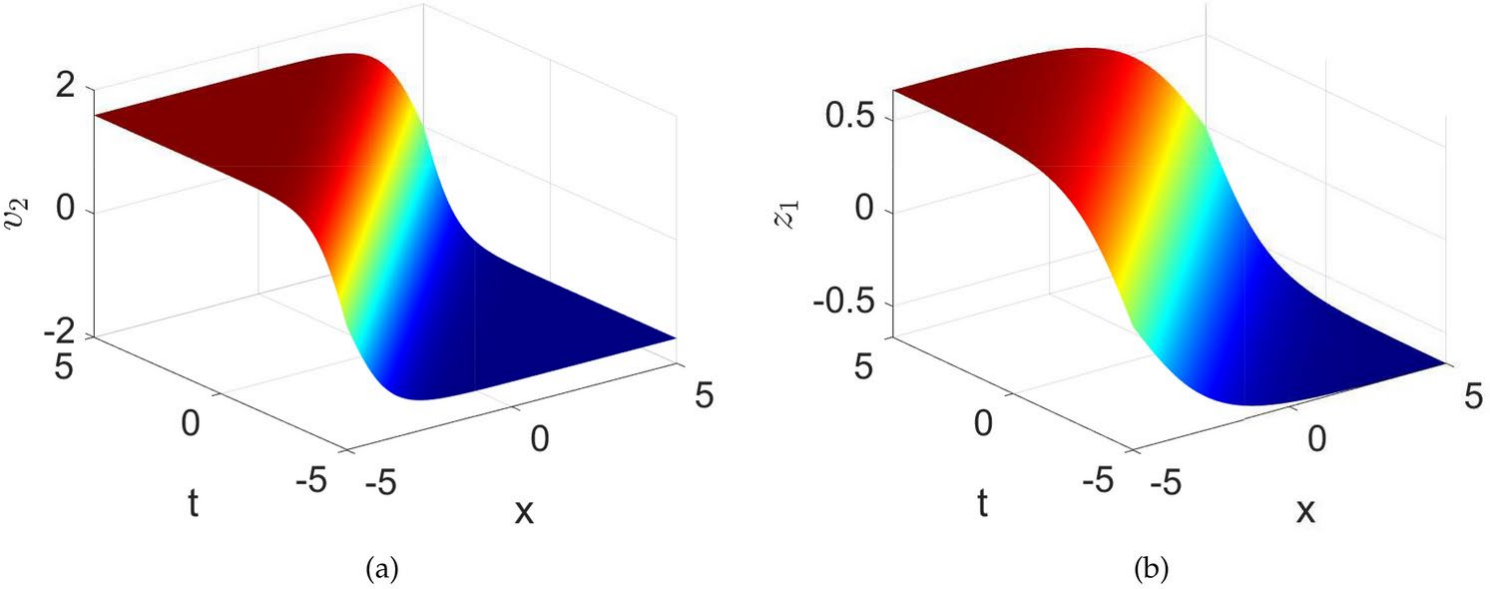
Numerical depiction of the exact solutions $\textsf{v}_{2}$ and $\textsf{z}_{1}$ presented in equations (31) and (32), respectively with parameters used as $\sigma =1,~C=-0.4,~k1=0.001,~\lambda =1,~\delta _{0}=0$.

The exact solutions $\textsf{v}_{2}$ and $\textsf{z}_{1}$ are graphically demonstrated in the Fig.13, where the kinks can be observed. These kink solitons portray a sharp transition between two different states of the magnetic field or magnetization within the material. The formation and propagation of kink solitons can arise from the interplay of nonlinear effects, such as exchange interactions, anisotropy, and magnetostatic interactions, in the presence of short-wave excitations.

When $\textsf{h}=0$, we obtain $\upsilon _{1\textsf{h}}^{2}=0$ and $\upsilon _{2\textsf{h}}^{2}=-\frac{2\mathcal {F}_{2}}{\mathcal {F}_{1}}$. So, one may achieve the following bright and dark solitons that adhere to the governing equation.

33 $$\begin{aligned} \textsf{v}_{3,4}\left( x,t\right)= & \pm \sqrt{-\frac{2\mathcal {F}_{2}}{\mathcal {F}_1}} sech\left( \sqrt{-\frac{\mathcal {F}_2}{1+\mathcal {F}_3}}\left( \Lambda x+\sigma t-\delta _0\right) \right) , \end{aligned}$$

34 $$\begin{aligned} \textsf{z}_{3,4}\left( x,t\right)= & \frac{1}{6\sigma }\left( \pm \sqrt{-\frac{2\mathcal {F}_{2}}{\mathcal {F}_1}} sech\left( \sqrt{-\frac{\mathcal {F}_2}{1+\mathcal {F}_3}}\left( \Lambda x+\sigma t-\delta _0\right) \right) \right) ^{3}\nonumber \\ & +\frac{\mathcal {C}}{\sigma }\left( \pm \sqrt{-\frac{2\mathcal {F}_{2}}{\mathcal {F}_1}} sech\left( \sqrt{-\frac{\mathcal {F}_2}{1+\mathcal {F}_3}}\left( \Lambda x+\sigma t-\delta _0\right) \right) \right) , \end{aligned}$$

**Fig. 14:**
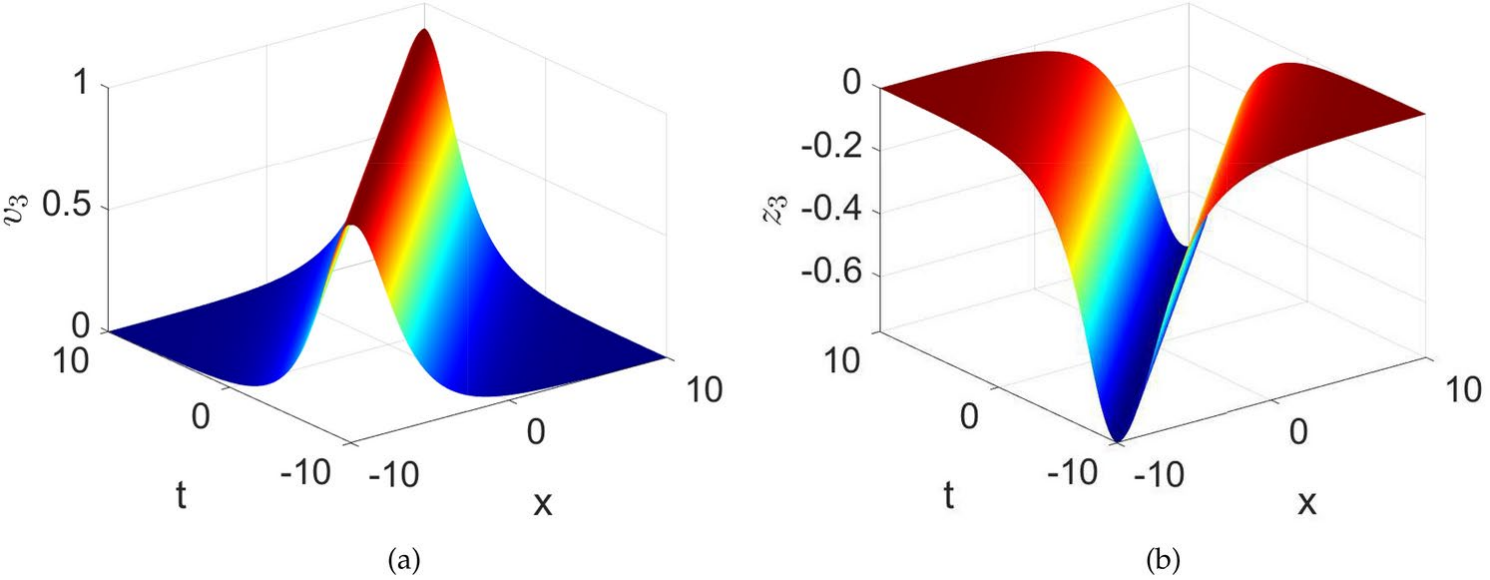
Numerical depiction of the exact solutions $\textsf{v}_{2}$ and $\textsf{z}_{1}$ presented in equations (33) and (34), respectively with parameters used as $\sigma =1,~C=-0.4,~k1=0.001,~\lambda =1,~\delta _{0}=0$.

The exact solutions $\textsf{v}_{3}$ and $\textsf{z}_{3}$ are numerically visualized in the Fig.14. Here it can be observed that the solution $\textsf{v}_{3}$, shows the bright solitary wave while the solution $\textsf{z}_{3}$ shows the dark solitary wave nature. Bright soliton could correspond to a localized region of increased magnetization or magnetic field intensity. This could arise due to nonlinear effects such as the self-focusing of magnetic waves or the formation of localized spin structures. Dark soliton could correspond to a localized region of reduced magnetization or magnetic field intensity. This could arise due to nonlinear effects such as the self-defocusing of magnetic waves or the formation of localized spin structures with opposite orientation compared to the surrounding medium.

## Periodic waves, bright and hybrid-bright solitary waves of the KMMS without damping

In this part of the manuscript, we method of a planar dynamic system is utilized to discover the periodic waves, bright and hybrid bright solitary waves that adhere to the KMMS without damping.

First, let us express Hamiltonian function of the (27) in the following form:

$$\begin{aligned} \mathcal {H}\left( \mathcal {V},\mathcal {W}\right) = \frac{\mathcal {W}^2}{2}-\frac{\mathcal {F}_1\mathcal {V}^4}{4}-\frac{\mathcal {F}_2\mathcal {V}^2}{2}=\textsf{h}, \end{aligned}$$

here $\textsf{h}$ denotes Hamiltonian constant. Since $\mathcal {F}_1>0$ and $\mathcal {F}_2>0$

When $\textsf{h}$ $\in$ $\left( \frac{\mathcal {F}_{2}^{2}}{4\mathcal {F}_1},0\right)$, as a result two different periodic orbits are obtained. Thus, we have:

35 $$\begin{aligned} \mathcal {W}^2= & \frac{\mathcal {F}_1}{2}\left( \mathcal {V}^4+\frac{2\mathcal {F}_2}{\mathcal {F}_1}\mathcal {V}^2+\frac{4\textsf{h}}{\mathcal {F}_1}\right) {,} \nonumber \\= & \frac{\mathcal {F}_1}{2}\left( \mathcal {V}^2-\upsilon _{1\textsf{h}^{2}}\right) \left( \upsilon _{2\textsf{h}^{2}}-\mathcal {V}^2\right) , \end{aligned}$$

where

$$\begin{aligned} \upsilon _{1\textsf{h}}=-\frac{\mathcal {F}_2}{\mathcal {F}_1}+\frac{\sqrt{\mathcal {F}^2_{2}-4\textsf{h}\mathcal {F}_1}}{\mathcal {F}_1}, \quad \upsilon _{2\textsf{h}}=-\frac{\mathcal {F}_2}{\mathcal {F}_1}-\frac{\sqrt{\mathcal {F}^2_{2}-4\textsf{h}\mathcal {F}_1}}{\mathcal {F}_1}. \end{aligned}$$

The insertion of Eq.(35) into first part of model (23) and then integrating periodic orbits, we get

$$\begin{aligned} \int _{-\upsilon _{2\textsf{h}}}^{\mathcal {V}}\frac{d\eta }{\sqrt{\left( \mathcal {\eta }^2-\upsilon _{1\textsf{h}^{2}}\right) \left( \upsilon _{2\textsf{h}^{2}}-\mathcal {\eta }^2\right) }}= & \pm \sqrt{\frac{\mathcal {F}_1}{2}}\left( \delta -\delta _0\right) , \\ \int _{\mathcal {V}}^{\upsilon _{2\textsf{h}}}\frac{d\eta }{\sqrt{\left( \mathcal {\eta }^2-\upsilon _{1\textsf{h}^{2}}\right) \left( \upsilon _{2\textsf{h}^{2}}-\mathcal {\eta }^2\right) }}= & \mp \sqrt{\frac{\mathcal {F}_1}{2}}\left( \delta -\delta _0\right) . \end{aligned}$$

Keeping in mind the preceding equation, one may obtain the subsequent solutions in terms of Jacobi function as:

36 $$\begin{aligned} \textsf{v}_{1,2}= & \pm \upsilon _{2\textsf{h}}dn\left( \upsilon _{2\textsf{h}}\sqrt{\frac{\mathcal {F}_1}{2}}\left( \Lambda x+\sigma t-\delta _0\right) , \frac{\sqrt{\upsilon _{2\textsf{h}}^2-\upsilon _{1\textsf{h}}^2}}{\upsilon _{2\textsf{h}}}\right) , \end{aligned}$$

37 $$\begin{aligned} \textsf{z}_{1,2}= & \frac{1}{6\sigma }\left( \pm \upsilon _{2\textsf{h}}dn\left( \upsilon _{2\textsf{h}}\sqrt{\frac{\mathcal {F}_1}{2}}\left( \Lambda x+\sigma t-\delta _0\right) , \frac{\sqrt{\upsilon _{2\textsf{h}}^2-\upsilon _{1\textsf{h}}^2}}{\upsilon _{2\textsf{h}}}\right) \right) ^{3}\nonumber \\ & +\frac{\mathcal {C}}{\sigma }\left( \pm \upsilon _{2\textsf{h}}dn\left( \upsilon _{2\textsf{h}}\sqrt{\frac{\mathcal {F}_1}{2}}\left( \Lambda x+\sigma t-\delta _0\right) , \frac{\sqrt{\upsilon _{2\textsf{h}}^2-\upsilon _{1\textsf{h}}^2}}{\upsilon _{2\textsf{h}}}\right) \right) , \end{aligned}$$

**Fig. 15:**
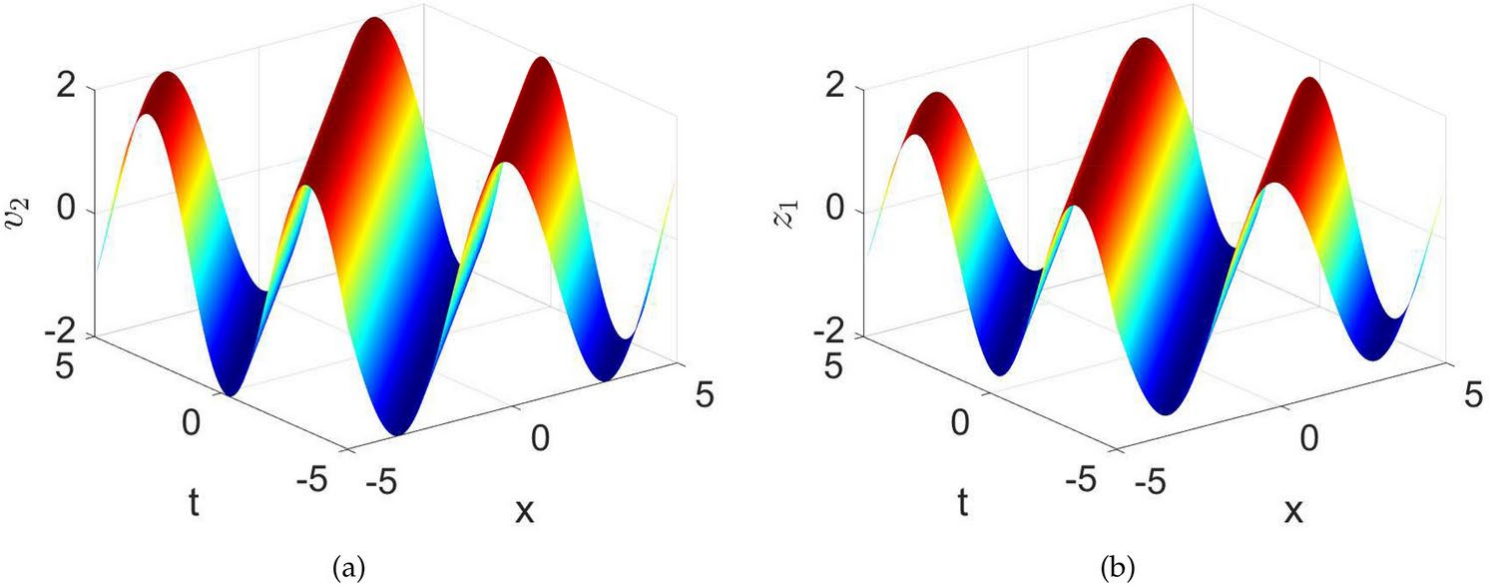
Numerical depiction of the exact solutions $\textsf{v}_{2}$ and $\textsf{z}_{1}$ presented in equations (36) and (37), respectively with parameters used as $\sigma =1,~C=-0.4,~k1=0.001,~\lambda =1,~\delta _{0}=0$.

The solutions $\textsf{v}_2$ and $\textsf{z}_{1}$ presented in (36) and (37) are respectively visualized in the Fig.(15). Here we see the periodic wave dynamics. The graph (15a) displays a periodic nature for the magnetization $\textsf{v}(x,t)$. The periodic nature of the solution elucidates that the wave propagating through the ferromagnetic material is stable and does not change its shape or amplitude over time. This is the feature of wave in a medium with no damping, where the energy is conserved, and the wave can travel indefinitely without weakening. The subgraph (15b) also shows the periodic nature of external magnetic field. Since there is no damping, the externel magnetic field does not experience any loss of energy either, allowing it to sustain the oscillations without decaying over time.

When $\textsf{h}=0$, we obtain $\upsilon _{1\textsf{h}}^{2}=0$ and $\upsilon _{2\textsf{h}}^{2}=-\frac{2\mathcal {F}{2}}{\mathcal {F}{1}}$. Therefore, we are able to form the following bright solitons that meet the requirements of the governing equation.

38 $$\begin{aligned} \textsf{v}_{3,4}\left( x,t\right)= & \pm \sqrt{-\frac{2\mathcal {F}_{2}}{\mathcal {F}_1}} sech\left( \sqrt{-\mathcal {F}_2}\left( \Lambda x+\sigma t-\delta _0\right) \right) ,\end{aligned}$$

39 $$\begin{aligned} \textsf{z}_{3,4}\left( x,t\right)= & \frac{1}{6\sigma }\left( \pm \sqrt{-\frac{2\mathcal {F}_{2}}{\mathcal {F}_1}} sech\left( \sqrt{-\mathcal {F}_2}\left( \Lambda x+\sigma t-\delta _0\right) \right) \right) ^{3}\nonumber \\ & +\frac{\mathcal {C}}{\sigma }\left( \pm \sqrt{-\frac{2\mathcal {F}_{2}}{\mathcal {F}_1}} sech\left( \sqrt{-\mathcal {F}_2}\left( \Lambda x+\sigma t-\delta _0\right) \right) \right) , \end{aligned}$$

**Fig. 16:**
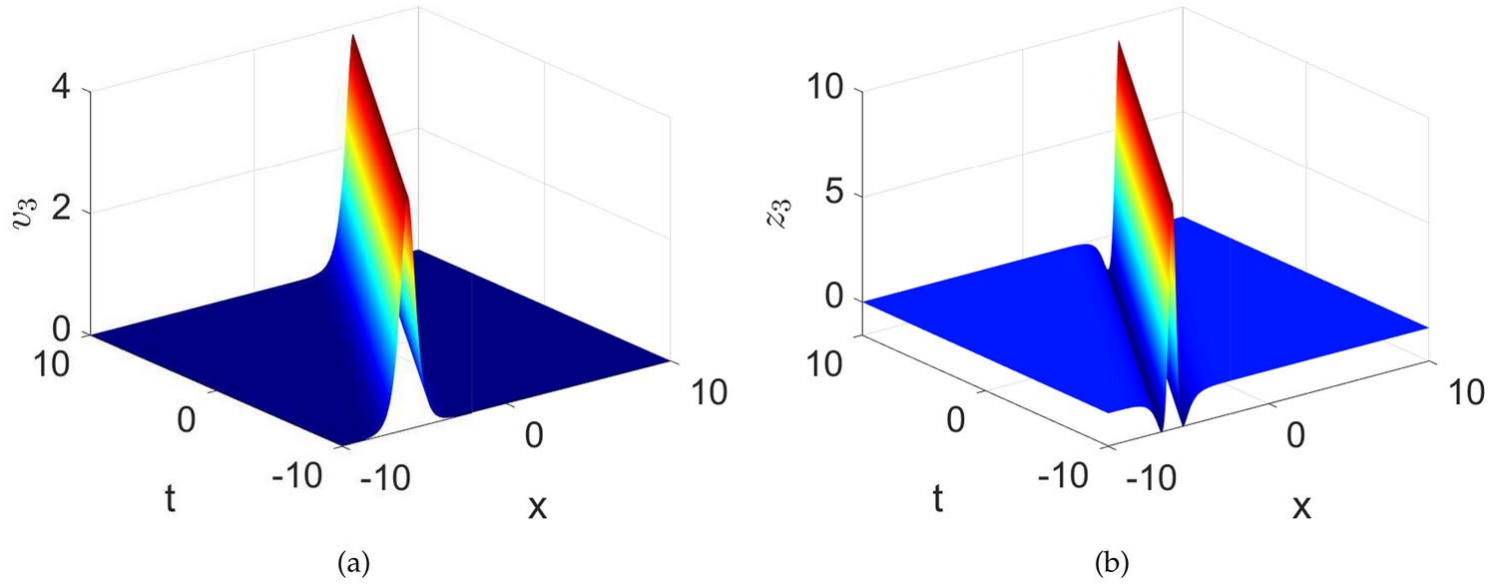
Numerical depiction of the exact solutions $\textsf{v}_{3}$ and $\textsf{z}_{3}$ presented in equations (38) and (39), respectively with parameters used as $\sigma =1,~C=-0.4,~k1=0.001,~\lambda =1,~\delta _{0}=0$.

The exact solutions $\textsf{v}_{3}$ and $\textsf{z}_{3}$ presented in equations (38) and (39) are numerically simulated in the Fig.16. One can observe that the solution $\textsf{v}_{3}$, shows the bright solitary wave while the solution $\textsf{z}_{3}$ shows hybrid-bright solitary wave nature.

## Conclusion

In this manuscript, we have investigated the Kraenkel-Manna-Merle (KMM) system, which models the nonlinear propagation of short waves in saturated ferromagnetic materials under an external field, even in the absence of conductivity. We employed two distinct methods to explore new solitary wave solutions for the KMM system. First, we applied the Lie group transformation to convert the KMM system into a system of nonlinear ordinary differential equations (ODEs). These ODEs were then solved analytically using the similarity invariant approach. The solutions obtained include bright, dark, and exponential solitary waves, which were visualized through 2D and 3D graphs. Second, we used wave and Galilean transformations to reduce the KMM system to a system of two ODEs, both with and without the damping effect. We conducted a detailed bifurcation analysis, sensitivity analysis, and examined the chaotic dynamics of the system. The results, displayed via graphs, reveal strange multi-scroll chaotic dynamics in the presence of damping and off-boosting dynamics in the absence of damping. Additionally, we applied the direct method and planar dynamical theory to obtain further new soliton solutions of the KMM system. These include bright, kink, dark, and periodic soliton solutions, which were also illustrated through 2D and 3D visualizations.

Beside this, some features like sensitivity, bifurcation, chaos and some soliton solutions of KMM system have been reported in the literature^45^. The authors have used Sardar subequation method to derive various types of solutions. However, here, we study Invariant solutions, Lie symmetry analysis, dynamical properties and some soliton solutions of KMM system for both with and without damping effect, via planar dynamical theory. Our findings have significant implications for applications in magnetic data storage, magnonic devices, material science, and spintronics, where these types of soliton solutions could play a crucial role. The optimal system for the Lie algebra has been used for analysis of a coupled system. This approach and some more advanced methods can be applied to the considered equation in the future work.

## Data Availability

All data generated or analysed during this study are included in this published article.

## References

[1] Ahmad, Jamshad et al. Soliton solutions of fractional extended nonlinear Schrödinger equation arising in plasma physics and nonlinear optical fiber. *Scientific Reports* **13**(1), 10877 (2023). 37407643 10.1038/s41598-023-37757-y PMC10322836

[2] Wazwaz, Abdul-Majid., Weaam, Alhejaili & El-Tantawy, S. . A. Study on extensions of (modified) Korteweg-de Vries equations: Painlevé integrability and multiple soliton solutions in fluid mediums. *Physics of Fluids* **35**(9), (2023).

[3] Ozisik, Muslum, Secer, Aydin & Bayram, Mustafa. On solitary wave solutions for the extended nonlinear Schrödinger equation via the modified F-expansion method. *Optical and Quantum Electronics* **55**(3), 215 (2023).

[4] Hussain, Akhtar et al. Exact solutions for the Cahn-Hilliard equation in terms of Weierstrass-elliptic and Jacobi-elliptic functions. *Scientific Reports* **14**(1), 13100 (2024). 38849360 10.1038/s41598-024-62961-9 PMC11637053

[5] Malik, Sandeep, Hashemi, Mir Sajjad, Kumar, Sachin, Hadi Rezazadeh, Mahmoud, W., & Osman, M. S. Application of new Kudryashov method to various nonlinear partial differential equations. *Optical and Quantum Electronics* **55**(1), 8 (2023).

[6] Shi, Ying, Zhang, Jia-man, Zhao, Jun-xiao & Zhao, Song-lin. Abundant analytic solutions of the stochastic KdV equation with time-dependent additive white Gaussian noise via Darboux transformation method. *Nonlinear Dynamics* **111**(3), 2651–2661 (2023).

[7] Zhao, Xin, Tian, Bo., Yang, Dan-Yu. & Gao, Xiao-Tian. Conservation laws, N-fold Darboux transformation, N-dark-bright solitons and the Nth-order breathers of a variable-coefficient fourth-order nonlinear Schrödinger system in an inhomogeneous optical fiber. *Chaos, Solitons & Fractals* **168**, 113194 (2023).

[8] Hussain, Akhtar, Hassan Ali, M., Usman, Zaman, F. D., & Park, Choonkil. Some New Families of Exact Solitary Wave Solutions for Pseudo-Parabolic Type Nonlinear Models. *Journal of Mathematics* 2024(1), 5762147 (2024).

[9] Biswas, Swapan, Ghosh, Uttam & Raut, Santanu. Construction of fractional granular model and bright, dark, lump, breather types soliton solutions using Hirota bilinear method. *Chaos, Solitons & Fractals* **172**, 113520 (2023).

[10] Ahmad, Shabir, Saifullah, Sayed, Khan, Arshad & Wazwaz, Abdul Majid. Resonance, fusion and fission dynamics of bifurcation solitons and hybrid rogue wave structures of Sawada-Kotera equation. *Communications in Nonlinear Science and Numerical Simulation* **119**, 107117 (2023).

[11] Zhu, Chaoyang, Mawaheb Al-Dossari, Rezapour, S., & Gunay, B. On the exact soliton solutions and different wave structures to the (2+ 1) dimensional Chaffee-Infante equation. *Results in Physics* **57**, 107431 (2024).

[12] Zhu, Chaoyang, Mawaheb Al-Dossari, S., Rezapour, S. Shateyi. & Gunay, B. Analytical optical solutions to the nonlinear Zakharov system via logarithmic transformation. *Results in Physics* **56**, 107298 (2024).

[13] Kai, Yue, Ji, Jialiang & Yin, Zhixiang. Study of the generalization of regularized long-wave equation. *Nonlinear Dynamics* **107**(3), 2745–2752 (2022).

[14] Kai, Yue & Yin, Zhixiang. Linear structure and soliton molecules of Sharma-Tasso-Olver-Burgers equation. *Physics Letters A* **452**, 128430 (2022).

[15] Niwas, Monika, Kumar, Sachin, Rajput, Rahi & Chadha, Dinsha. Exploring localized waves and different dynamics of solitons in (2+ 1)-dimensional Hirota bilinear equation: a multivariate generalized exponential rational integral function approach. *Nonlinear Dynamics* 1–14 (2024).

[16] Naowarat, Surapol, Saifullah, Sayed, Ahmad, Shabir & De la Sen, Manuel. Periodic, singular and dark solitons of a generalized geophysical KdV equation by using the tanh-coth method. *Symmetry* **15**(1), 135 (2023).

[17] Khaliq, Saqib, Aman Ullah, Shabir Ahmad, Ali Akgül, Abdullahi Yusuf, & Sulaiman, Tukur A. “Some novel analytical solutions of a new extented (2+ 1)-dimensional Boussinesq equation using a novel method.” Journal of Ocean Engineering and Science (2022).

[18] Ahmad, Shabir, Gafel, Hanan S., Khan, Aizaz, Khan, Meraj Ali, & ur Rahman, Mati. Optical soliton solutions for the parabolic nonlinear Schrödinger Hirota’s equation incorporating spatiotemporal dispersion via the tanh method linked with the Riccati equation. *Optical and Quantum Electronics* **56**(3), 382 (2024).

[19] Kumar, Sachin & Hamid, Ihsanullah. New interactions between various soliton solutions, including bell, kink, and multiple soliton profiles, for the (2+ 1)-dimensional nonlinear electrical transmission line equation. *Optical and Quantum Electronics* **56**(7), 1–24 (2024).

[20] Li, Zai-Dong, Qiu-Yan Li, Lu Li, & W. M. Liu. “Soliton solution for the spin current in a ferromagnetic nanowire.” Physical Review E 76(2): 026605 (2007). 10.1103/PhysRevE.76.026605 17930165

[21] Wang, Kang-Jia., Shi, Feng & Wang, Guo-Dong. Abundant Soliton Structures to the (2+1)-Dimensional Heisenberg Ferromagnetic Spin Chain Dynamical Model. *Advances in Mathematical Physics* **2023**, 1–9 (2023).

[22] Ma, Yu-Lan. & Li, Bang-Qing. Kraenkel-Manna-Merle saturated ferromagnetic system: Darboux transformation and loop-like soliton excitations. *Chaos, Solitons & Fractals* **159**, 112179 (2022).

[23] Kraenkel, Roberto André, Manna, M. . A. & Merle, V. Nonlinear short-wave propagation in ferrites. *Physical Review E* **61**(1), 976 (2000). 10.1103/physreve.61.976 11046355

[24] Li, Bang-Qing. & Ma, Yu-Lan. Rich soliton structures for the Kraenkel-Manna-Merle (KMM) system in ferromagnetic materials. *Journal of Superconductivity and Novel Magnetism* **31**, 1773–1778 (2018).

[25] Li, Bang-Qing. & Ma, Yu-Lan. Loop-like periodic waves and solitons to the Kraenkel-Manna-Merle system in ferrites. *Journal of Electromagnetic Waves and Applications* **32**(10), 1275–1286 (2018).

[26] Mohammed, Wael W., El-Morshedy, M., Cesarano, Clemente & Al-Askar, Farah M. Soliton solutions of fractional stochastic Kraenkel-Manna-Merle equations in ferromagnetic materials. *Fractal and Fractional* **7**(4), 328 (2023).

[27] Alshammari, Mohammad, Hamza, Amjad E., Cesarano, Clemente, Aly, Elkhateeb S. & Mohammed, Wael W. The analytical solutions to the fractional Kraenkel-Manna-Merle system in ferromagnetic materials. *Fractal and Fractional* **7**(7), 523 (2023).

[28] Raza, Nauman et al. New and more dual-mode solitary wave solutions for the Kraenkel-Manna-Merle system incorporating fractal effects. *Mathematical Methods in the Applied Sciences* **45**(5), 2964–2983 (2022).

[29] Sahoo, Subhadarshan & Saha Ray, S. Lie symmetry analysis and exact solutions of (3+ 1) dimensional Yu-Toda-Sasa-Fukuyama equation in mathematical physics. *Computers & mathematics with applications.* **73**(2), 253–260 (2017).

[30] Bihlo, Alexander, & Popovych, Roman O. “Lie symmetry analysis and exact solutions of the quasigeostrophic two-layer problem.” Journal of Mathematical Physics 52, no. 3 (2011).

[31] Bai, Yu-Shan., Liu, Ya.-Na. & Ma, Wen-Xiu. Lie symmetry analysis, exact solutions, and conservation laws to multi-component nonlinear Schrödinger equations. *Nonlinear Dynamics* **111**(19), 18439–18448 (2023).

[32] Luo, Renfei, Abbas, Naseem, Hussain, Akhtar & Ali, Shahbaz. A new sensitive visualization, solitary wave profiles and conservation laws of ion sound waves arising in plasma. *Optical and Quantum Electronics* **56**(3), 415 (2024).

[33] Kumar, Sachin & Dhiman, Shubham Kumar. Exploring cone-shaped solitons, breather, and lump-forms solutions using the lie symmetry method and unified approach to a coupled breaking soliton model. *Physica Scripta.* **99**(2), 025243 (2024).

[34] Kumar, Sachin, Kaur, Lakhveer & Niwas, Monika. Some exact invariant solutions and dynamical structures of multiple solitons for the (2+ 1)-dimensional Bogoyavlensky-Konopelchenko equation with variable coefficients using Lie symmetry analysis. *Chinese Journal of Physics* **71**, 518–538 (2021).

[35] Kumar, Sachin, Dhiman, Shubham Kumar & Chauhan, Astha. Analysis of Lie invariance, analytical solutions, conservation laws, and a variety of wave profiles for the (2+ 1)-dimensional Riemann wave model arising from ocean tsunamis and seismic sea waves. *The European Physical Journal Plus* **138**(7), 1–22 (2023).

[36] Kumar, Sachin, Ma, Wen-Xiu., Dhiman, Shubham Kumar & Chauhan, Astha. Lie group analysis with the optimal system, generalized invariant solutions, and an enormous variety of different wave profiles for the higher-dimensional modified dispersive water wave system of equations. *The European Physical Journal Plus* **138**(5), 434 (2023).

[37] Hussain, Akhtar et al. Dynamics of invariant solutions of the DNA model using Lie symmetry approach. *Scientific Reports* **14**(1), 11920 (2024). 38789463 10.1038/s41598-024-59983-8 PMC11126696

[38] Usman, Muhammad, Hussain, Akhtar, Zidan, Ahmed M. & Mohamed, Abdullah. Invariance properties of the microstrain wave equation arising in microstructured solids. *Results in Physics* **58**, 107458 (2024).

[39] Hussain, Akhtar, Usman, Muhammad & Zaman, Fiazuddin. Lie group analysis, solitons, self-adjointness and conservation laws of the nonlinear elastic structural element equation. *Journal of Taibah University for Science* **18**(1), 2294554 (2024).

[40] Hussain, Akhtar, Zaman, F. D., Owyed, Saud, Herrera, Jorge & Sallah, Mohammed. Analyzing invariants and employing successive reductions for the extended Kadomtsev Petviashvili equation in (3+ 1) dimensions. *PloS one* **19**(7), e0305177 (2024). 38954677 10.1371/journal.pone.0305177 PMC11218999

[41] Zhu, C., Al-Dossari, M., Rezapour, S., Alsallami, S. A. M. & Gunay, B. Bifurcations, chaotic behavior, and optical solutions for the complex Ginzburg-Landau equation. *Results in Physics* **59**, 107601 (2024).

[42] Li, Zhao & Huang, Chun. Bifurcation, phase portrait, chaotic pattern and optical soliton solutions of the conformable Fokas-Lenells model in optical fibers. *Chaos, Solitons & Fractals* **169**, 113237 (2023).

[43] Ahmad, Shabir, Lou, Jie, Ali Khan, Meraj & ur Rahman, Mati. Analysing the Landau-Ginzburg-Higgs equation in the light of superconductivity and drift cyclotron waves: Bifurcation, chaos and solitons. *Physica Scripta* **99**(1), 015249 (2023).

[44] Khan, Arshad, Saifullah, Sayed, Ahmad, Shabir, Ali Khan, Meraj & ur Rahman, Mati. Dynamical properties and new optical soliton solutions of a generalized nonlinear Schrödinger equation. *The European Physical Journal Plus* **138**(11), 1059 (2023).

[45] Borhan, J. R. M., Mamun Miah, M., Alsharif, Faisal & Kanan, Mohammad. Abundant Closed-Form Soliton Solutions to the Fractional Stochastic Kraenkel-Manna-Merle System with Bifurcation, Chaotic, Sensitivity, and Modulation Instability Analysis. *Fractal and Fractional* **8**(6), 327 (2024).

